# N_2_O Decomposition on Singly and Doubly (K and Li)-Doped Co_3_O_4_ Nanocubes—Establishing Key Factors Governing Redox Behavior of Catalysts

**DOI:** 10.1021/jacs.4c06587

**Published:** 2024-08-23

**Authors:** Leszek Nowakowski, Camillo Hudy, Filip Zasada, Joanna Gryboś, Witold Piskorz, Anna Wach, Yves Kayser, Jakub Szlachetko, Zbigniew Sojka

**Affiliations:** † Faculty of Chemistry Jagiellonian University, ul. Gronostajowa 2, Krakow 30-387, Poland; ‡ National Synchrotron Radiation Centre SOLARIS Jagiellonian University, ul. Czerwone Maki 98, Kraków 30-392, Poland; § Physikalisch-Technische Bundesanstalt (PTB), Abbestr. 2-12, Berlin 10587, Germany; ∥ Doctoral School of Exact and Natural Sciences, Jagiellonian University, Prof. St. Łojasiewicza St 11, Krakow 30-348, Poland

## Abstract

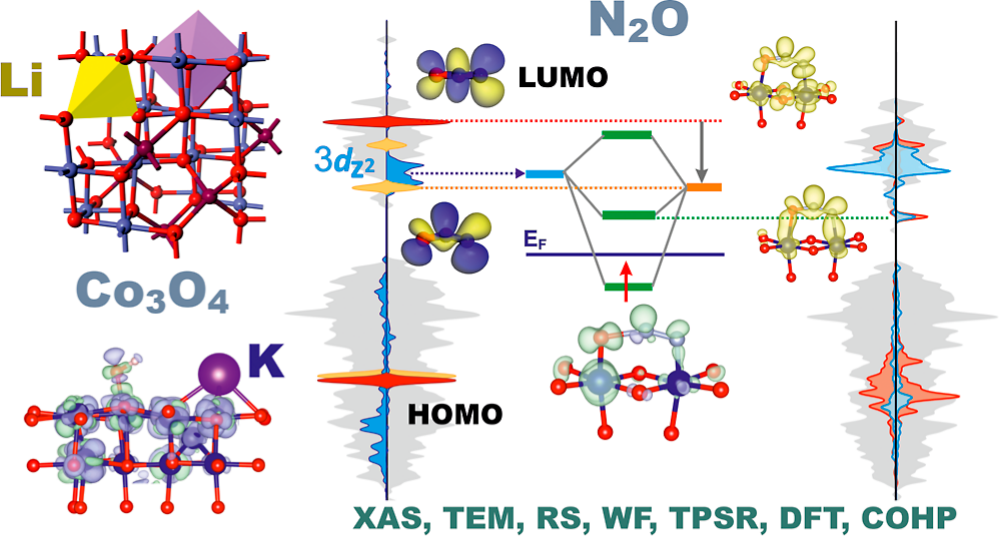

The intimate mechanism of N_2_O decomposition on bare and redox-tuned Co_3_O_4_ nanocubes (achieved by single (Li or K) and double (Li and K) doping) was elucidated. The catalysts synthesized by the hydrothermal method were characterized by X-ray electron absorption fine structure measurements, X-ray diffraction, Raman spectroscopy, scanning electron microscopy, transmission electron microscopy, and Kelvin Probe techniques. TPSR and steady-state isothermal catalytic tests reveal that the N_2_O turnover frequencies are critically sensitive to the work function of the catalysts, adjusted purposely by doping. For the catalysts obtained by one-pot hydrothermal synthesis, lithiation of the Co_3_O_4_ nanocubes leads to the formation of {Li’_8a_, Co·_16d_} species, decreasing steadily the work function and the activity, while for the catalysts prepared by postsynthesis impregnation, formation of {Li’_8a_, Co’_16d,_ Co··_16c_} species leads to a volcano-type dependence of the catalytic activity and the work function in parallel. The beneficial effect of potassium was discussed in terms of mitigation of surface potential buildup due to the accumulation of ionosorbed oxygen intermediates (surface electrostatics), which hinders the interfacial electron transfer. Analysis of the catalytic activity response to the redox tuning of Co_3_O_4_, substantiated by DFT calculations, allowed for a straightforward conceptualization of the redox nature of the N_2_O decomposition in terms of the lineup of frontier orbitals of the N_2_O/N_2_O^–^ and O_2_^–^/O_2_ reactants with the surface DOS structure and the resultant molecular orbital interactions. The positions of the virtual bonding 3π_g_^0^(N_2_O)−α-3d_*z*2_ and the occupied 2π_g_^1^(O_2_^–^)−α-3d_*z*2_ states relative to the Fermi energy level play a crucial role in the regulation of the forward and backward interfacial electron transfer events, which drive the redox process.

## 1 Introduction

Catalytic decomposition of N_2_O has been widely studied over simple and mixed oxides,^1−5^ ABO_3_ perovskites,^6,7^ AB_2_O_4_ spinels,^8−10^ hydrotalcite-derived oxides,^11,12^ mesoporous silica materials,^13^ zeolites,^14−16^ and a variety of supported catalysts.^17−20^ Among them, cobalt spinel catalysts belong to one of the most promising systems for fundamental and applied investigations.^21−23^ Depending on the synthesis conditions, cobalt spinel (Co_3_O_4_) can be easily obtained in the form of well-shaped cubic, octahedral, or cuboctahedral nanocrystals,^24^ and its composition modified via substitution at *A* and *B* sites, providing excellent materials for rigorous fundamental investigations into the structure-redox reactivity relationships, using N_2_O decomposition as a convenient model process.^1,2,25,26^

Substantial improvement in the redox performance of the catalyst may be achieved by bulk structure modification (doping with alien cations)^1,2,27−29^ or by tuning the electronic properties with alkali promoters.^26,30−33^ The latter effect is usually significant and results in a lowering of the reaction temperature of N_2_O decomposition even by 200 °C.^1,2,25^ In our previous articles, we have shown that such beneficial action is mainly of electronic origin and is associated with the presence of alkali adspecies (K, Cs)^25,26,33^ but not with their diffusion into the catalyst bulk as previously suggested.^27^ The alkali promoters present on the catalyst surface, by lowering the work function of the cobalt spinel, facilitate the forth and back interfacial electron transfer processes that occur between the spinel active sites and the N_2_O reactant (dissociative reduction step) and the reaction of the O_2_^–^_(ads)_/O_2_^2–^_(ads)_ intermediates (oxidative oxygen evolution step). Although the activation energy of the decomposition of nitrous oxide over potassium or cesium-promoted cobalt spinel is fairly well correlated with changes in the work function of the catalyst,^2,25,26,34^ the intimate molecular nature of this process and its relationship with the redox properties of the catalyst have not been elucidated in the required detail so far.

Incorporation of the nonredox dopant ions into the spinel matrix provides another way of tuning the electronic properties of the catalyst on purpose.^2,35^ The framework positions 8a (tetrahedral) and 16c (octahedral) or interstitial 16d (octahedral) and 48f (tetrahedral) are the possible loci for such substitution (for details, see Electronic Supporting Information, Figure S1, and the associated text). This has allowed for straightforward identification of the cobalt active site due to the selective removal of Co^2+^ in the 8a positions by doping with redox inert Mg^2+^ cations or replacement of Co^3+^ in the 16c positions with Al^3+^.^35^ Valence pinning of cobalt cations can be achieved in turn by aliovalent substitution. In this context, lithium ions, due to their small radius, are particularly suitable species to be incorporated.^36−38^ The presence of hypovalent Li cations in the spinel matrix gives rise to an alternation of the cobalt oxidation state imposed by a charge compensation constraint, modifying the catalyst Fermi level energy (*E*_F_) and the associated catalytic properties.^2,39−42^

The inherent intricacy of the interfacial redox processes, resulting from the complex DOS structure of the semiconducting oxide catalyst, and a variety of alignment patterns between the energy bands of the active sites and frontier (HOMO–LUMO) orbitals of the reactants, makes a comprehensive, in-depth understanding of all involved molecular events still a challenging endeavor. As a functional approach, several redox reactivity descriptors have been proposed (particularly in accounting for the ORR and OER electrocatalytic activity), including *e*_g_ level filling, antibonding orbital occupancy, the density of the states near the Fermi level, and the d-band center or position of the highest occupied d-state vs the Fermi level.^43−46^ In the field of thermal oxide catalysis, the establishment of analogous descriptors is still less advanced.^47−49^ However, by realizing the complex nature of the interfacial redox processes, the validity of such singular parameters focused on only the catalyst component is of rather limited value for establishing a more general account. In this context, the relative energies of the electronic levels of the reactants/intermediates and the catalyst active sites along with the corresponding molecular orbital coupling strengths play a pivotal role.

A concise epitomization of the heterogeneous redox process in terms of the electronic structure of both interacting moieties (catalyst surface DOS structure and the frontier molecular orbital (FMO) pattern of the reactant admolecules), taking into account the corresponding orbital interactions (p*-*d hybridization), the effects resulting from the position of the Fermi level, and variable surface electrostatics in particular, has not been achieved as yet.

The catalytic gas/solid redox processes are inherently associated with interfacial forth electron transfer (reduction of reactants/intermediates) and back electron transfer (oxidation of reactants/intermediates) events. Due to intimate contact between the reaction pair, allowing for sizable orbital interactions, they usually proceed along the inner-sphere, adiabatic routes featured by strong orbital coupling.^50^ In such electron transfer processes, bonds are often formed and broken, and semiclassical approaches based, e.g., on the candid Gerischer model,^51^ cannot be used directly for interpretation of the redox behavior of the involved catalyst/reactant couples.

Despite the apparent mechanistic simplicity of the N_2_O decomposition over oxide catalysts,^1,2,52^ the kinetics of this reaction can often be complex, or even elusive, due to a varying number of the active sites with temperature, the presence of the charged O_*n*_^z–^_(ads)_ intermediates, and mixed reaction-surface diffusion kinetics.^53−56^ Therefore, it may exhibit unexpectedly intriguing characteristics regarding, e.g., a wide variation of the activation energies values (Δ*E*_a_), reported even when nominally the same catalysts (such as cobalt spinel) are used.^1,2,57−59^

In the present study, we address these points for catalytic N_2_O decomposition, which served as a useful redox probe reaction, choosing an euhedral cobalt spinel and its doped derivatives as a dedicated model catalytic system. The aim is to evaluate the redox behavior of cobalt spinel catalysts of a well-defined nanocubic shape and unravel the effects resulting from bulk and surface doping of Co_3_O_4_ with two different alkali cations (lithium and potassium), introduced alone or jointly. The resulting controlled electronic structure perturbations of the cobalt spinel allow for straightforward clarification of the observed changes in the catalytic activity in terms of the Fermi level and surface potential variation (due to the accumulation of the O_*n*_^z–^_(ads)_ intermediates on the catalyst surface), playing along with the substantial interfacial orbital interactions a central function.

The constructed conceptual model of N_2_O decomposition on the cobalt spinel *p*-type semiconductor catalysts rationalizes the mechanism of the interfacial back and forth electron transfer events, which govern the course of this redox reaction. Due to the focus on the redox nature of the deN_2_O reaction only, the effect of typical contaminants (O_2_, H_2_O, and NO) was intentionally omitted, as it has been thoroughly elucidated for cobalt spinel-based catalysts in our previous paper.^60^

## 2 Experimental Section

### 2.1 Catalysts Synthesis

Cube-shaped Co_3_O_4_ nanoparticles were synthesized by the template-free hydrothermal method, described previously by us.^24,61^ For this purpose, Co(NO_3_)_2_·6H_2_O and NaOH precursors with the molar ratio 2:1 were dissolved in 10 mL of deionized H_2_O and transferred to a 20 mL Teflon-lined steel autoclave. The reaction mixture was heated at 180 °C for 5 h and then cooled down to room temperature. The obtained black precipitate was separated by centrifugation, washed several times with distilled water to remove sodium ions, and dried at 60 °C in air overnight (this sample is labeled as h-Co_3_O_4_). The final product was annealed at 500 °C in the air for 5 h to form monodispersed cobalt spinel nanocubes of high crystallinity and submicrometer size (c-Co_3_O_4_ samples). The lithium-doped h-Li_*x*_Co_3–*x*_O_4_ series (0.040 ≤ *x* ≤ 0.099) was obtained via an analogous one-pot hydrothermal synthesis by adding NaOH along with the LiOH dopant in the 2:1, 1:1, and 1:2 molar ratios to the reaction mixture before the hydrothermal reaction. For the synthesis of the i-Li_*x*_Co_3_O_4_ (0.033 ≤ *x* ≤ 0.060) and i-K_*y*_/Li_0.045_Co_3_O_4_ (0.0013 ≤ *y* ≤ 0.012) series, the calcined c-Co_3_O_4_ nanocubes were next doped with lithium and potassium by incipient wetting impregnation using aqueous solutions of LiNO_3_ or KNO_3_, respectively. The impregnated spinels were finally calcined at 600 (for the Li doping step) and 450 °C (for the K doping step). Following the literature, after such treatment, the small Li cations are incorporated into the spinel matrix,^62^ whereas the larger potassium ions remain on the surface.^2,25^ An auxiliary series of the lithiated samples with an extended Li/Co content ranging from 3.29 × 10^–3^ to 2.31 × 10^–1^ was obtained by dry impregnation of Co_3_O_4_ (obtained by precipitation with NaOH^33,35^) using aqueous solutions of lithium nitrate of appropriate concentrations.

### 2.2 Methods

X-ray diffraction patterns were recorded with a Rigaku Miniflex X-ray diffractometer in the 2θ range of 15–85° with a resolution of 0.02° and a time interval of 1s per step. The chemical composition of lithium-doped samples was determined by inductively coupled plasma mass spectrometry (ICP-MS, PerkinElmer with a FIAS system). For this purpose, the spinel sample was dissolved in concentrated HCl (∼35%).

The loading of potassium deposited on the surface of the spinel catalysts was assessed by means of energy-dispersive X-ray spectrometry (XRF, Thermo Scientific, ARL QUANT’X), equipped with the Rh anode operating at an acceleration voltage of 4–50 kV (1 kV steps) and beam size of 1 mm.

The μ-Raman spectra were recorded in the range of 100–900 cm^–1^ with 1 cm^–1^ resolution using a Renishaw InVia spectrometer, equipped with the CCD detector and a confocal Leica DMLM microscope, with an excitation wavelength of 514 nm. Nine scans were accumulated for each measurement to achieve a satisfactory signal-to-noise ratio. Scanning electron microscopy (SEM) imaging of the spinel nanocrystals’ morphology was performed on a Tescan VEGA 3 equipped with a LaB_6_ cathode. The samples were gold-coated before the microscopic observations. Scanning transmission electron microscopy (STEM) imaging of the samples was performed with a FEI Tecnai/Osiris electron microscope equipped with an X-FEG Schottky emitter (200 kV). For energy dispersive X-ray (EDX) mapping, a 4-sector silicon drift windowless detector and the Bruker Esprit software were used. Prior to the microscopic analyses, the samples were deposited on a lacey carbon film supported on a copper grid (Agar Scientific, London, UK, 300 mesh).

Contact potential difference (CPD) measurements were carried out by the Kelvin dynamic condenser method with a KP6500 probe (McAllister Technical Services). To standardize the catalyst surface, the measurements were carried out in vacuum (10^–7^ mbar) at 150 °C after annealing the samples at 400 °C. The work function values, Φ, were calculated from the relation: CPD = Φ_reference_ – Φ_sample_, using a standardized stainless steel plate as a reference (*d* = 3 mm, Φ_reference_ = 4.3 eV).

X-ray absorption fine structure (XAFS) measurements in the soft X-ray energy range were performed at the plane grating monochromator beamline of Physikalisch-Technische Bundesanstalt at the electron storage ring BESSY II. The XAFS spectra were collected in the fluorescence mode after installing the samples on a goniometer in an ultrahigh vacuum chamber equipped with a windowless silicon drift detector, which was oriented at 90° and in the polarization plane of the synchrotron radiation.^63^ The samples were oriented at 45° to the incidence direction and with the normal vector to the sample surface plane contained within the polarization plane of synchrotron radiation. The X-ray beam spot size on the sample was around 0.14 mm^2^. The Co L2,3-edge XAFS spectra were acquired in the 750–830 eV energy range with energy increments of 500 meV between data points. The OK-edge spectra were collected in the energy range of 500 to 600 eV with the same energy increments. The XAFS spectra were normalized to the 0 to 1 range for subsequent analysis. The X-ray absorption spectra in the hard X-ray energy range were collected on a laboratory spectrometer. The setup was composed of an X-ray source (XOS X-Beam Superflux PF with a Mo anode) and a von Hámos geometry-based X-ray spectrometer to detect the X-ray radiation transmitted through the sample. The spectrometer was adjusted to diffract radiation by the Si(110) crystal in the diffraction order of *n* = 4 and detect the signal on the two-dimensional X-ray camera (Andor Newton DO920P). The size of the X-ray beam spot on the sample was 100 μm × 100 μm. The energy calibration was performed numerically by finding the best fit of the reference spectrum (from the XAS database) to the measured spectrum of a cobalt foil, assuming a linear pixel-to-energy conversion function. The recorded spectra were normalized in the 0–1 range.

### 2.3 Catalytic Measurements

The isothermal and temperature-programmed (TPSR) catalytic N_2_O decomposition tests were performed in the 25–600 °C range using a homemade setup operating in the LabView environment and equipped with a Hiden Analytical HPR20 QMS detector, Brooks mass controllers, and automatic Valco switching valves. The experiments were carried out using a quasi-CSTR-type quartz flow reactor and 150 mg of the catalyst (sieve fraction of 0.2–0.3 mm). The feed flow of 42–102 mL·min^–1^ and the heating rate of 10 °C·min^–1^ were used. The N_2_O conversion was calculated based on the QMS signals normalized against the helium gas balance as
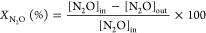
1whereas the reaction turnover frequencies, TOF_N_2_O_, were calculated using the equation
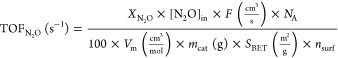
2where *F* = 30 mL/min is the flow rate, *V*_m_ = 22,400 cm^3^ is the gas molar volume (*T* = 298 K, *p* = 1 bar), *m*_cat_ = 0.15 g is the catalyst mass, *S*_BET_ is its specific surface area, and *n*_surf_ = 6 × 10^18^ 1/m^2^ is the surface concentration of Co^3+^ cations on the (100) facet.^64^ The reproducibility of the catalytic tests was checked by performing 3 consecutive measurements of N_2_O decomposition on bare Co_3_O_4_, Li-, K-, and (Li,K)-doped cobalt spinel catalysts in the TPSR mode. The obtained N_2_O conversion profiles were virtually identical (see Figure S10 in the ESI Section). The accuracy of activation energy determination was assessed using standard error analysis of the slope in the Arrhenius equation. The apparent reaction order (*m* = ∂ ln *r*_N_2_O_/∂ ln *p*_N_2_O_) was determined from the pressure dependence of the reaction rate (*r*_N_2_O_ = *kp*_N_2_O_^*m*^).

### 2.4 Molecular Modeling

DFT calculations were carried out using the VASP code and the projector-augmented plane wave method (PAW).^65^ The preliminary geometry optimization was performed at the PB/DFT + *U* level of theory with the Hubbard parameter *U* = 3.5 eV applied to the Co-3d states,^66,67^ whereas the electronic structure calculations were performed with the hybrid HSE06 functional. Typically, we used the standard Monkhorst–Pack^68^ grid with the 5 × 5 × 5 sampling mesh for the bulk and the 3 × 3 × 1 mesh for slab calculations; the cutoff energy was set to 500 eV, and the SCF convergence criterion was set to 1 × 10^–5^ eV. For the atomic position relaxation, the conjugate-gradient method, improved by Brent’s steps corrector algorithm, was employed,^69^ within the convergence criterion of 1 × 10^–4^ eV·Å^–1^. The population analysis was carried out by means of the Bader method. The bulk cobalt spinel unit cell was obtained by fully optimizing all internal degrees of freedom of the cubic (1 × 1 × 1) cell containing 56 ions (Co_24_O_32_). For the modeling of the localized electronic states, the occupation matrix control method (OCC-MAT) was used.^70^ The work function of the parent and doped catalysts was calculated using slab models of different sizes (1 × 1, 2 × 1, and 3 × 1 for modeling the dopant loading effect), with 11 Å of the oxide thickness (for details, see Figure S2 in the ESI Section). The atomic positions in the four top and four bottom layers of the slab models were relaxed within the criterion of 1 × 10^–4^ eV·Å^–1^. The applied vacuum spacing was sufficiently large for the successful convergence of the planar averaged electrostatic potential to a constant value far from the surface, taken as the reference vacuum level (Figure S3 in ESI). Projected crystal orbital Hamilton population (pCOHP) was analyzed using the Lobster v5.0 package.^71^ This method allows for partitioning the band-structure energy into bonding, nonbonding, and antibonding contributions. The integrated projected crystal orbital hamilton population (IpCOHP) calculated up to the Fermi level serves to quantify the strength of the chemical bonds between the involved atomic moieties. Following the Newns-Anderson type treatment, the lifetime of the activated adspecies was estimated from the halfwidth of the chemisorption (hybridization) function approximated by the Lorentzian shape as τ = (η/2)Δ^–1^.^72^

## 3 Results and Discussion

### 3.1 Characterization of the Catalyst

To determine the critical value of Li doping of Co_3_O_4_ that preserves the spinel phase homogeneity (lack of undesired, spurious LiCoO_2_), we examined at first an auxiliary series of the lithiated samples with the extended Li/Co content ranging from 3.29 × 10^–3^ to 2.31 × 10^–1^ (see Table S1 in ESI). As revealed by the X-ray diffraction (XRD) and Raman spectra (Figure S4), the spinel phase is preserved for Li/Co < 4 × 10^–2^. Above this threshold value, gradual formation of the surface LiCoO_2_ phase was observed, as revealed by the development of the (006) and (104) diffraction peaks (see Figure S4b_1_,b_2_, respectively). The formation of the LiCoO_2_ phase was also confirmed by the appearance of bands at 490 and 595 cm^–1^, assigned to the E_g_ and A_1g_ vibrations, respectively (Figure S4c). As a result, for further investigations, we restricted the Li/Co ratio below the ascertained critical limit since the LiCoO_2_ phase appeared completely inactive in the N_2_O decomposition in comparison to the Co_3_O_4_ phase, as revealed in a reference experiment (see Figure S4d).

A list of the spinel pure phase samples doped by Li and K (the prime series), used for further investigation, and their compositions determined by ICP-MS (Li) and XRF (K) techniques are shown in Tables S2 and S3 in the ESI Section. In the case of potassium, noting its accommodation on the surface (see below), its areal atomic concentration, *n*_K_, was calculated based on the BET surface area value.

**Figure 1:**
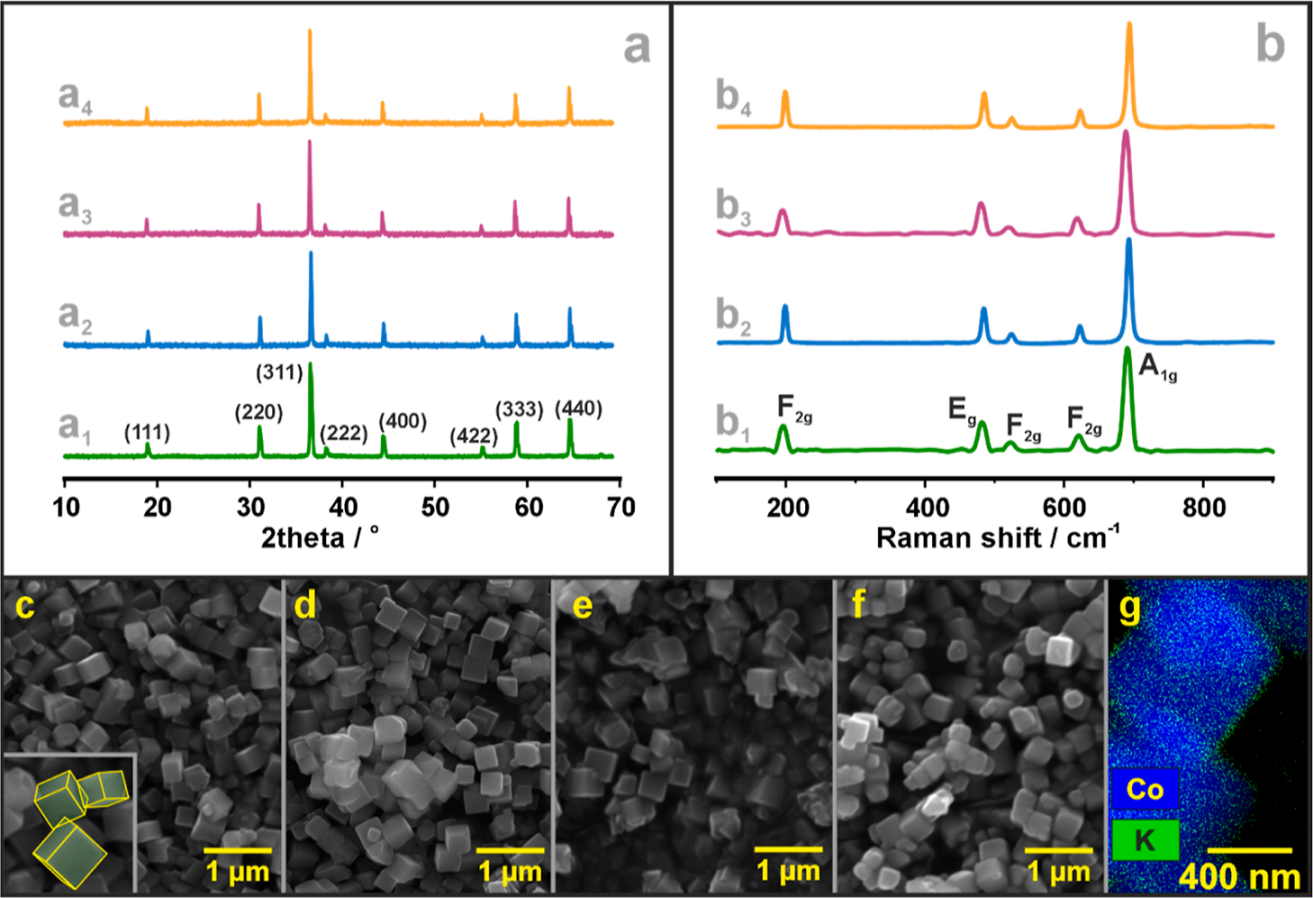
Selected XRD patterns (a) of the parent Co_3_O_4_ (a_1_—green line), i-Li-2-Co (a_2_—blue), h-Li-2-Co (a_3_—purple), and i-K-5/i-Li-2-Co samples (a_4_—yellow), and the corresponding Raman spectra (b_1_–b_4_) together with SEM pictures (c–f). Exemplary STEM/EDX map of the potassium distribution in the i-K-5/i-Li-2-Co sample (g). The results of the characterization of the remaining samples are shown in Figures S5 and S6 in ESI.

The phase purity of the parent cobalt spinel and the Li and K-doped catalysts was verified by XRD and Raman spectroscopic measurements (Figures 1, S5 and S6). The diffraction patterns (Figures 1a and S5a, S6a) show that the spinel catalysts obtained are highly crystalline (all the observed XRD lines can be indexed within the *Fd*3̅*m* space group), 24210-ICSD, and no segregated phases comprising Li or K were identified in the applied doping range. Their possible presence in high surface dispersions (preventing XRD detection) was also excluded by Raman measurements (Figures 1b, S5b, and S6b). The Raman spectra of the Co_3_O_4_, i-Li_*x*_Co_3_O_4_, h-Li_*x*_Co_3–*x*_O_4_, i-K_*y*_/Co_3_O_4_, and i-K_*y*_/Li_0.045_CoO_4_ samples are all characterized by 5 bands located at 194 (F_2g_), 480 (E_g_), 520 (F_2g_), 620 (F_2g_), and 690 (A_1g_) cm^–1^,^73^ confirming good purity and high crystallinity of the singly and doubly doped spinel catalysts.

The selected SEM pictures (Figure 1c–f), representative for the given series of the catalyst, reveal that the synthesized spinel nanocrystals exhibit clear-cut cubic morphology (see reconstructed shapes in the inset in Figure 1c), with a size of 780 ± 60 nm (gauged by the body diagonal), regardless of the presence or absence of the dopants and the way along which they were introduced.

STEM/EDX mapping confirmed accumulation of potassium essentially on the surface of the spinel nanocubes for both singly K- and doubly (K,Li)-promoted catalysts, as expected for the postsynthesis impregnation applied for potassium deposition and the fact that the large ionic radius prevents its effective incorporation into the spinel matrix. Exemplary map of K-distribution for the most active i-K-5/i-Li-2-Co catalyst is shown in Figure 1g.

For lithium doping via one-pot hydrothermal synthesis of the catalysts, the Li cations are directly incorporated into the spinel matrix in the course of the reaction. Whatever is the framework location of the lithium ions (in the tetrahedral 8a or octahedral 16d sites), in this case, the parent cobalt cations always become oxidized to maintain the charge balance. Since disclosing the actual Li locus by the applied spectroscopic methods appeared elusive (due to the detection limitations), we established the preferred tetrahedral 8a site of lithium localization by means of DFT modeling, applying the occupational matrix (OCC-MAT) scheme (see below).

The impregnation of the Co_3_O_4_ nanocubes with the LiNO_3_ solution and subsequent calcination were monitored by using QMS and Raman techniques. In the temperature range of 300–450 °C, an evolvement of NO and O_2_ (with a small amount of NO_2_) was observed due to the decomposition of the nitrate anions (Figure S7). The incorporation of the Li^+^ cations into the spinel lattice was confirmed by a systematic hypsochromic shift of the Raman A_1g_ band with an increasing lithium content (Figure S8). There are two possible scenarios of this process depending on the mechanistic subtleties of the adsorbed nitrate decomposition. When this step is concomitant with the substitution of Li^+^ for the tetrahedral Co^2+^ cations, the latter are shifted from the framework 8a into the empty interstitial octahedral 16c positions.^74^ This is accompanied by a reduction of the Co^3+^ cations located at the 16d sites and a commensurate evolution of NO and O_2_ in equal amounts
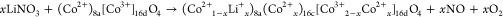
3as observed in Figure S7, indeed.

In an alternative mechanism of lithium incorporation, the nitrate precursor is first decomposed into Li_2_O adspecies, with an equal formation of the NO, O_2_, and NO_2_ gas products.
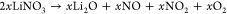
4a

The resultant *x*Li_2_O entities are next integrated with the spinel host, and the Li^+^ ions entering the 8a sites oxidize the Co^3+^ cations in the 16d positions to maintain the charge balance.
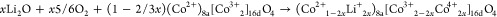
4b

In the first case, Li doping is tantamount to the formation of two Li’_8a_ and Co’_16d_ species, which are charge-balancing the interstitial Co··_i_ centers. In the second case, the Li’_8a_ defects are balanced by the hole Co·_16d_ centers, as in the case of the one-pot synthesis. The evolution of small amounts of NO_2_ (Figure S7) speaks in favor of the Co^3+^ reduction described in eq 3. Nevertheless, to ascertain which of these two possibilities actually takes place, we performed X-ray absorption spectroscopy (XAFS) investigations into the alterations of the cobalt oxidation state upon the lithiation. It should be emphasized, however, that in both cases Li^+^ cations are located in the tetrahedral (8a) sites in the spinel matrix, but the cobalt valence pinning effects imposed by the disparate routes of their incorporation are different.

**Figure 2:**
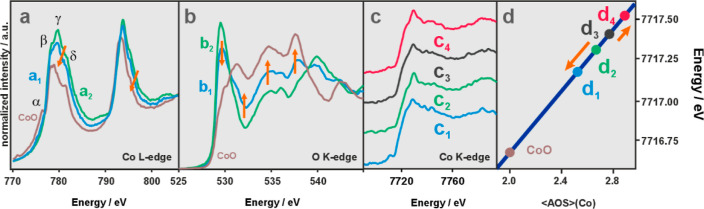
Co L-edge (a), O K-edge (b), and Co K-edge XAFS spectra (c) together with the cobalt <AOS> correlation diagram (d) for i-Li_0.045_Co_3_O_4_ (a_1_–d_1_), c-Co_3_O_4_ (a_2_–d_2_), h-Co_3_O_4_ (c_3_,d_3_) h-Li_0.086_Co_2.94_O_4_ (c_4_,d_4_), and the reference CoO samples.

The redox and structural changes induced by Li doping were examined using the oxygen K, and cobalt L and K-edges for the bare Co_3_O_4_, and the most active (among the lithiated spinels) catalyst i-Li-2-Co (i-Li_0.045_Co_3_O_4_), see Figure 2a,b. The collected Co L-edge spectra show two main regions, lower energy L_3_-edge and higher energy L_2_-edge, which arise from transitions of 2p_3/2_ → 3d and 2p_1/2_ → 3d, respectively. The L_2,3_-edge spectra provide rich electronic structure information, being highly sensitive to valency, spin states, and coordination geometries.^75^ Generally, the recorded XAFS Co L-spectrum of the Li-impregnated spinel exhibits clear features (marked by orange arrows) indicative of a partial reduction of Co^3+^ to Co^2+^. This observation is further confirmed by the analysis presented in Figure 2b, where the differences between the O K-edge spectra of the CoO, Co_3_O_4_, and i-Li_*x*_Co_3_O_4_ samples are directly compared. The peak position of the O K-edge at 531 eV, assigned to O 1s → O 2p transitions,^76^ remains essentially intact for the c-Co_3_O_4_, i-Li_*x*_Co_3_O_4_, and CoO samples, as expected for the unchanged oxidation state of the O^2–^ anions, but its intensity is significantly reduced upon Li doping. Together with the associated more dramatic changes in the postedge region, these variations speak in favor of the incorporation of lithium into the cobalt spinel according to eq 3 when introduced via impregnation. Inspection of the Co L_2,3_-edges properly confirmed this finding. The γ and δ features in the L_3_-edge, assigned to Co^3+^ in the octahedral low spin coordination, decrease in line with the fact that the content of the Co^3+^_16d_ (*S* = 0) cations is reduced upon Li doping (feature α corresponds to high spin octahedral Co^2+^, characteristic of CoO),^77^ [and references therein]. The concomitant decline and broadening of the β features, due to high spin tetrahedral Co^2+^, are consistent with the presence of Li cations in the 8a sites, resolving the incorporation mechanism definitely.

The cobalt K-edge spectra of Co_3_O_4_ and CoO show a significant energy shift between the valence states 2+ and 3+, allowing reliable elucidation of the cobalt average oxidation state in the Li-doped spinel catalysts, based on the position of the inflection point in the recorded K-edge spectra (Figure 2c). The resulting average oxidation state values of cobalt, <AOS>, for the bare h-Co_3_O_4_ (<AOS> = 2.76) and c-Co_3_O_4_ (<AOS> = 2.67) samples, and the lithiated i-Li_0.045_Co_3_O_4_ (<AOS> = 2.55) and h-Li_0.086_Co_2.94_O_4_ (<AOS> = 2.89) catalysts are shown in Figure 2d.

Enhancement of the <AOS> values of cobalt upon calcination of the h-Co_3_O_4_ catalyst obtained by the hydrothermal method results from the annihilation of the cationic vacancies (the missing surface Co^2+^ cations), which is accompanied by oxygen release, as previously shown by us.^78^ The calcination process, Co_1–*x*_Co_2_O_4_ →(1 – *x*)Co_3_O_4_ + 2*x*O_2_, leads to a decrease in the <AOS> value from 8/(3 – *x*) = 2.76 to 8/3, as observed in the XAFS spectra (Figure 2c,d). Thus, the composition of the hydrothermally obtained cobalt spinel nanocubes (h-Co_3_O_4_) corresponds actually to , whereas the calcined c-Co_3_O_4_ nanocubes assume the stoichiometric composition with <AOS> = 2.67. The determined cobalt <AOS> values for the Li-doped samples definitely confirm that in the case of the impregnated lithium catalysts (i-Li_*x*_Co_3_O_4_), part of the octahedral Co^3+^ cations is reduced to Co^2+^, which is accounted for by eq 3. In contrast, for the lithiated cobalt spinel obtained via one-pot hydrothermal synthesis (h-Li_*x*_Co_3–*x*_O_4_), a fraction of the Co^3+^ cations is oxidized into Co^4+^, following eq 4a. As a result, both cobalt spinels that are lithiated in different ways exhibit dissimilar redox features, which can be easily controlled by the way Li is introduced.

**Figure 3:**
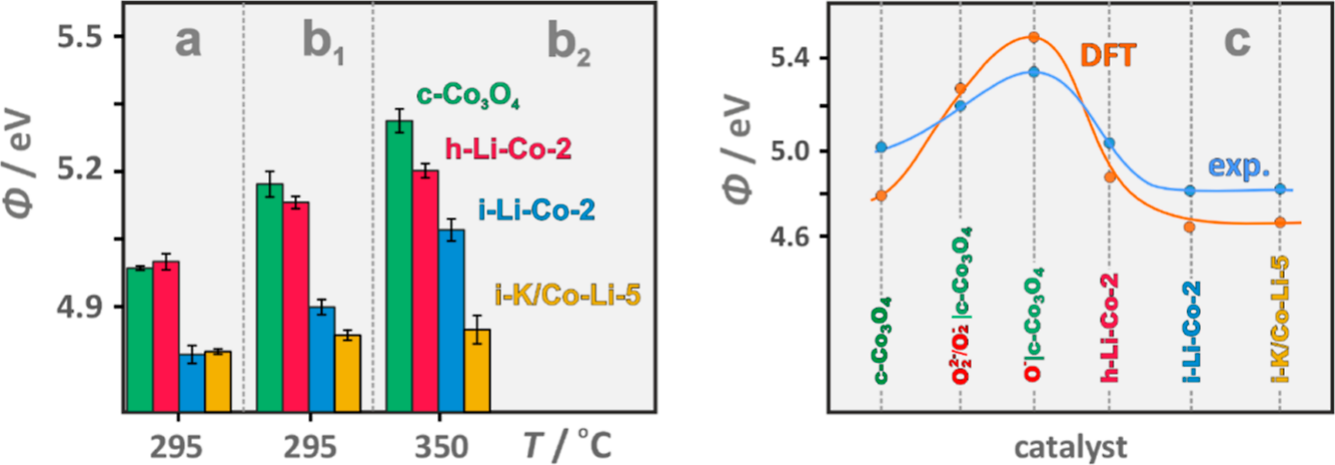
Work function measurements for bare c-Co_3_O_4_, singly doped h-Li-2-Co and i-Li-2-Co, and doubly doped i-K-5/Li-2-Co spinel samples measured in vacuum (a) and in the presence of O_2_ at 295 °C (b_1_) and 350 °C (b_2_). Comparison of the experimental (blue line) and DFT-calculated (orange line) work function variations for the investigated catalysts (c).

The work function, Φ, measurements in a vacuum (Figure 3a) and in oxygen (reaction product of N_2_O decomposition) at 295 and 350 °C (Figure 3b_1_,b_2_) were next performed to characterize the redox properties of the bare (c-Co_3_O_4_) and selected lithiated (h-Li-2-Co and i-Li-2-Co) samples, along with the doubly promoted catalyst (i-K-5/Li-2-Co) of the highest deN_2_O activity among all the investigated samples (see below). Variation in the work function with the K and Li loading is shown in Figure S9 (ESI Section). In both cases, a volcano-type behavior of Φ as a function of the lithium or potassium content was observed. The nonmonotonous changes in the work function for surface K doping are typically observed, and can well be accounted for by the Topping model^2^ [and references therein]. The origin of the minimum in the work function for the lithiated samples (Figure S9b) can be associated with the reduction of cobalt in the low concentration range (see Figure 2d), followed by the gradual development of LiCoO_2_ surface entities as the Li content increases above the critical value (see Figure S4).

For the impregnated catalysts, the presence of Co^2+^ in octahedral positions is well reflected by the lowering of the work function from Φ = 4.98 eV (c-Co_3_O_4_) down to 4.79 eV (i–Li-Co-2) and to 4.8 eV for the i-K-5/Li-2-Co catalysts when measured in a vacuum. For h-Li-2-Co, a slight work function enhancement to 5.04 eV remains in line with the formation of octahedral Co^4+^ holes. The Φ values for the bare c-Co_3_O_4_ and the lithiated spinels distinctly increase (ΔΦ = 0.19–0.1 eV) when measured at 295 °C in the presence of dioxygen, i.e., the reactant molecule of the highest electron affinity (EA = 0.45 eV^79^). On the contrary, for the doubly doped catalyst, the corresponding change was significantly smaller (ΔΦ = 0.03), the reason for which is explained below. When passing to a higher temperature (350 °C), the ΔΦ values augment further to ΔΦ = 0.33–0.2 and 0.04 eV for the singly and doubly doped catalysts, respectively.

In analogy to the previous treatment,^80^ the observed variation of the work function can be discussed in terms of the following factors: Φ = −*E*_F_ + *e*Δ*V*^0^_S_ + ΔΦ_dip_. The last two terms gauge the electrostatic barrier for electron removal to the vacuum level of zero energy, caused by the inherent surface potential of the bare surface (Δ*V*^0^_S_), and the Helmholtz surface potential created by the charged (ionosorbed) oxygen adspecies (ΔΦ_dip_ = *e* θ_ads_ μ_dip_/εε_0,_ where μ_dip_ is the magnitude of the surface dipole, θ_ads_ is the areal density of anionic oxygen adspecies, and ε and ε_0_ is the electric permittivity of the environment and vacuum, respectively^81^).

The formation, electronic structure, and stability of the negatively charged reactive oxygen adspecies, such as O_2_^–^ and O_2_^2–^ on the (100) surface of cobalt spinel below 295 °C, were previously described by us in detail.^61^ An electron acceptor character of the adsorption gives rise to the formation of surface dipoles (ΔΦ_dip_) with the protruding negative charge, leading to the observed enhancement of the work function, as reported elsewhere.^82,83^ The observed increase in the Φ values upon rising the temperature to 350 °C, in turn, results from partial dissociation of the diatomic O_2_^z–^ into the monatomic O^–^ adspecies of a larger dipole moment (see below).^82,84^ The much smaller sensitivity of the work function changes to the oxygen presence in the case of the doubly doped i-K-5/Li-2-Co catalyst can easily be explained by the formation of O–K^δ+^ surface dipoles of an inversed polarity, which counterbalance the surface potential produced by O_*n*_^z*–*^ adspecies.

As a result, by proper selection of the lithium doping technique, the redox state of the cobalt cations can intentionally be tuned owing to the valence pinning effect. The changes in the electronic structure and electrostatic state of the spinel surface upon doping and adsorption of the electron acceptor molecules, ascertained by XAFS and work function measurements, were next accounted for by DFT modeling. The experimental work function values are reasonably well reproduced by the calculation results (see Figure 3c), confirming definitely the proposed interpretation.

### 3.2 Catalytic Performance

For probing the redox behavior of the bare and Li/K-doped spinels, a catalytic N_2_O decomposition was used as a suitable model reaction. Neglecting, for the sake of simplicity, its involved mechanism (see Chapter S4 in ESI for more details), the reaction proceeds in two essential steps of prime dissociative reduction, epitomized as 2N_2_O + 2e^–^ → 2O^–^_ads_ + 2N_2(g)_, and successive oxidative oxygen evolution reaction, epitomized as 2O^–^_ads_ → O_2(g)_ + 2e^–^,^2,25,31,52^ probing directly the redox properties of the catalyst (e^–^ stands for the electron of the Fermi level energy). Such account remains in accordance with the apparent reaction order of 0.95–0.83 (see Figure S12c, and the associated discussion), where the leading reaction consists of the kinetically most demanding N_2_O dissociation. Its course is, however, significantly influenced by the subsequent oxygen evolution process, deviating the apparent reaction order from unity. Noteworthily, though both molecular events are sensitive to the applied doping, the overall reaction mechanism is still maintained, and the first step remains the rate-determining.

**Figure 4:**
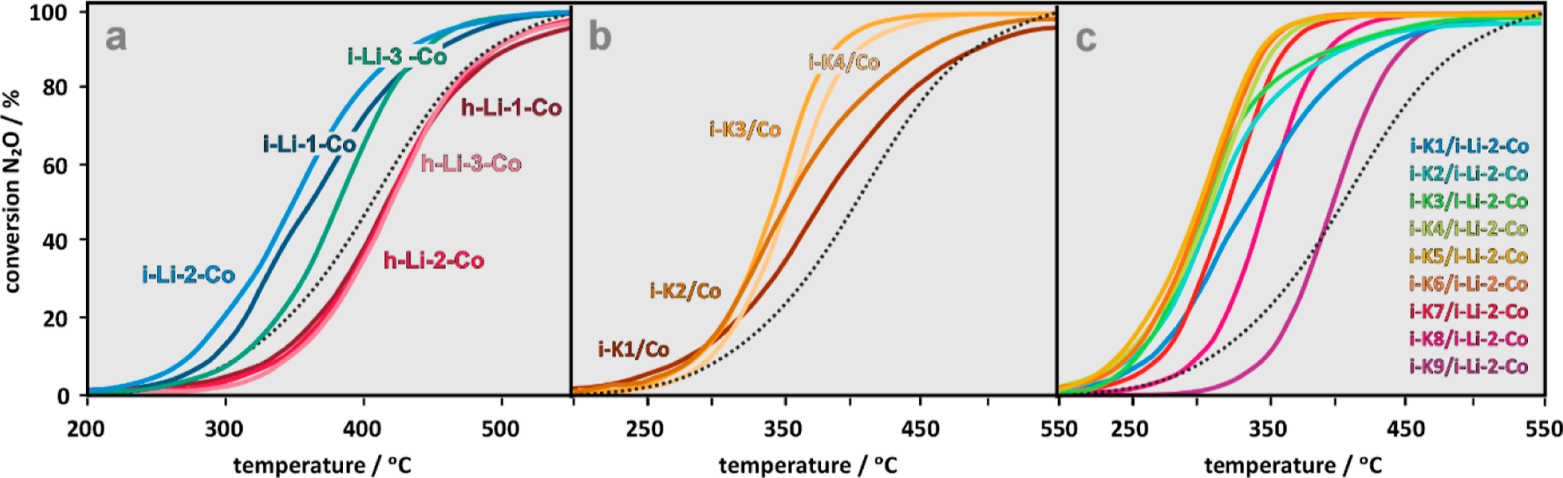
TPSR conversion curves of the N_2_O decomposition for the lithiated cobalt spinel catalysts obtained via impregnation (i-Li_*x*_Co_3_O_4_ series) and one-pot hydrothermal synthesis (h-Li_*x*_Co_3-x_O_4_ series) (a), together with the profiles of the potassium impregnated (i-K_*y*_/Co_3_O_4_ series) (b) and the Li and K impregnated catalysts (i-K_*y*_/Li_0.045_Co_3_O_4_ series) (c). The dotted lines correspond to the reference bare c-Co_3_O_4_ catalyst.

The N_2_O conversion curves for the lithiated Co_3_O_4_ samples measured in the TPSR mode as a function of the Li doping level are shown in Figure 4a. In the case of the i-Li_*x*_Co_3_O_4_ series, a nonmonotonous dependence of the N_2_O conversion on the lithium content was found, with the optimal value for the i-Li-2-Co sample. All of the catalysts in this series were more active compared to the parent c-Co_3_O_4_, in contrast to the h-Li_*x*_Co_3–*x*_O_4_ catalysts, which are noticeably less active and also less sensitive to the doping level. The corresponding Arrhenius plots are collated in Figure S11, along with the variation of the activation energy with an increasing concentration of lithium. While for the h-Li_*x*_Co_3–*x*_O_4_ catalysts, the Δ*E*_a_ values increase steadily with the Li doping level, in the case of the i-Li_*x*_Co_3_O_4_ series, it passes through a well-pronounced minimum for the i-Li-2-Co sample, revealing a dramatically opposite catalytic behavior in response to the spinel redox tuning, which is governed by the route the lithium dopant was introduced and its concentration (Figure S11a’).

**Figure 5:**
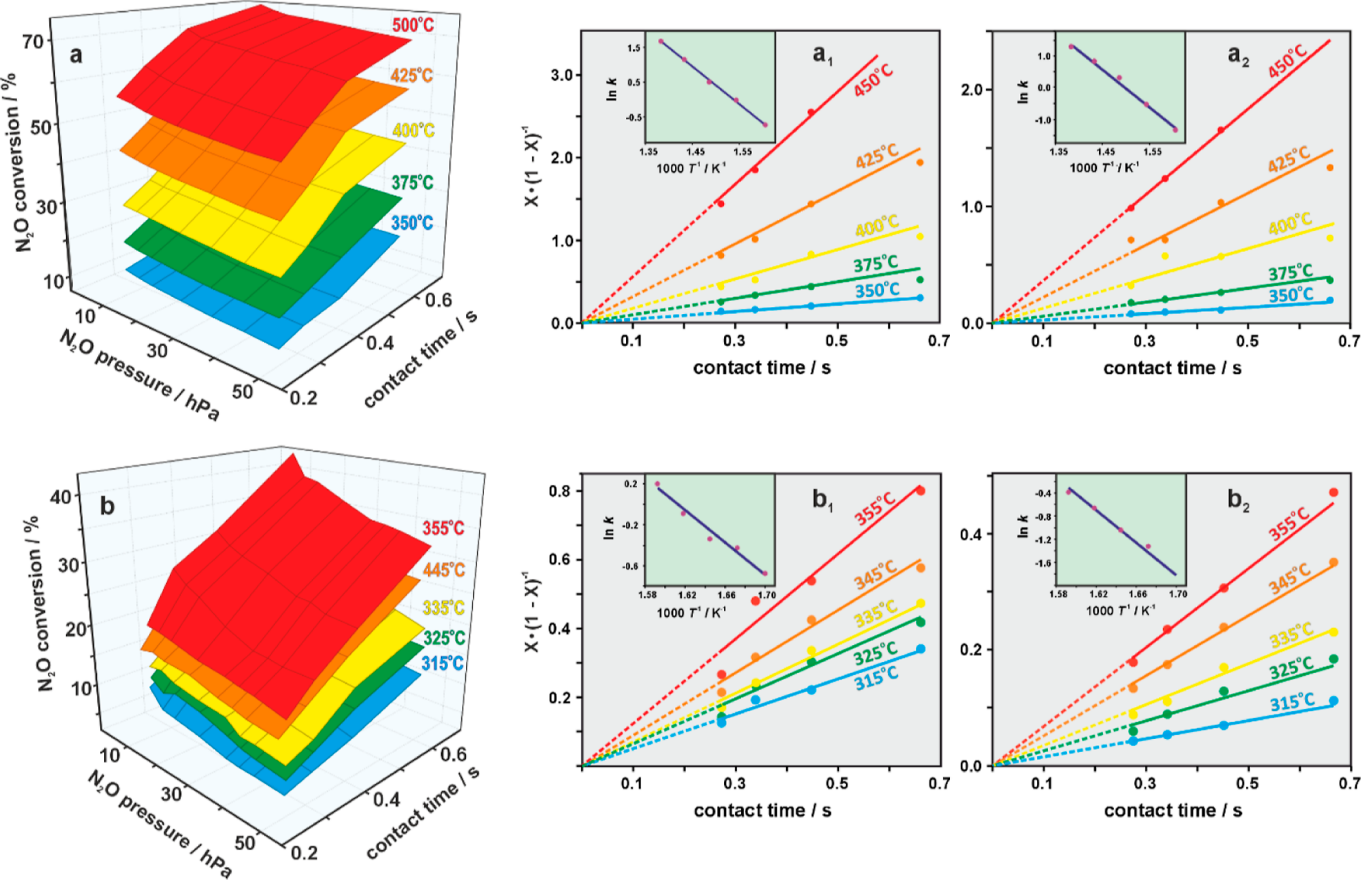
N_2_O conversion as a function of *p*(N_2_O) and the contact time for c-Co_3_O_4_ (a) and for the most active impregnated i-Li-2-Co (i-Li_0.045_Co_3_O_4_) catalyst (b). The corresponding *X*/(*X* – 1) plots are versus the contact time at various temperatures at *p*_N_2_O_ = 0 Pa (a_1_,b_1_) and 50 hPa (a_2_,b_2_). The associated Arrhenius plots are shown in the insets.

For the most active catalyst i-Li-2-Co and the reference c-Co_3_O_4_, the conversion dependence on the N_2_O pressure, *p*_N_2_O_, and on the contact time, τ, was additionally measured under the steady-state conditions (Figure 5a,b) at various temperatures (300 °C–500 °C). These results allow for accurate determination of the rate constants, *k*, and their temperature dependence at various surface coverages controlled by *p*_N_2_O_. According to our previous studies,^56,60^ within the mean-field approximation, a simple relation, *k*(*T*)·τ = *X*(*T*)[1 – *X*(*T*)], can be used for this purpose, for the type of the applied quasi-CSRT reactor. Next, a more detailed analysis of the temperature (and the surface coverage) dependence of the rate constants, *k*(*T*)_θ_, was performed for the two limiting cases corresponding to *p*_N_2_O_ = 50 hPa (θ = max in the investigated pressure range) and *p*_N_2_O_ → 0 hPa (θ = 0). The latter values were determined by extrapolating the pressure dependence of the conversion at constant contact time and temperature to 0 hPa (see Figure S12a,b). This approach allows for the determination of the intrinsic conversion value at zero surface coverage (*X*^0^, θ = 0), and the N_2_O conversion value, *X*^θ^_,_ at the surface coverage corresponding to the selected pressure, *p*_N_2_O_ = 50 hPa. These values may then easily be converted to the rate constants *k*(*T*)_θ_, as described above. The corresponding plots are shown in Figure 5a_1_,a_2_,b_1_,b_2_ for bare and lithiated Co_3_O_4_ (i-Li-2-Co), respectively.

The activation energies, Δ*E*_a_(*p*_N_2_O_), determined from the Arrhenius plots (see the corresponding insets in Figure 5a_1_,a_2_,b_1_,b_2_) for *p*_N_2_O_ = 50 hPa and *p*_N_2_O_ → 0 hPa are equal to Δ*E*_a_ = 1.03 ± 0.05 and 0.96 ± 0.04 eV for c-Co_3_O_4_ and Δ*E*_a_ = 1.16 ± 0.07 and 0.66 ± 0.07 eV for the i-Li-2-Co catalyst, respectively. Thus, in the case of the most active i-Li-2-Co catalyst, there is a striking sensitivity of the activation energy value to the N_2_O pressure, which is almost doubled when passing from *p*_N_2_O_ → 0 to 50 hPa. The corresponding changes in the activation energy for the reference bare cobalt spinel are apparently less pronounced. Such large variations in Δ*E*_a_ for N_2_O decomposition, repeatedly encountered in the literature,^1,2,57,58^ can be associated with the formation of negatively charged oxygen intermediates. The resultant buildup of a surface electrostatic field strongly influences the dynamics of the interfacial electron transfer processes. The latter constitute the very nature of the N_2_O decomposition redox, and this issue is addressed in more detail in the next section.

The effect of potassium doping on cobalt spinel activity is shown in Figure 4b. The observed nonmonotonous behavior of the catalyst performance as a function of the potassium loading is in agreement with our previous finding.^2,25^ It is associated with similar changes in the work function (see Figure S9a), which are well accounted for by the Topping equation.^2^ The influence of potassium doping on the catalytic performance of the best lithiated sample (i-Li-2-Co) is shown in Figure 4c. There is a pronounced synergy resulting from double doping with the most active catalyst, i-K-5/i-Li-2-Co, where the full conversion of N_2_O is already reached at *T* < 350 °C, while for the reference bare Co_3_O_4_ at this temperature, the conversion still remains below 20%.

**Figure 6:**
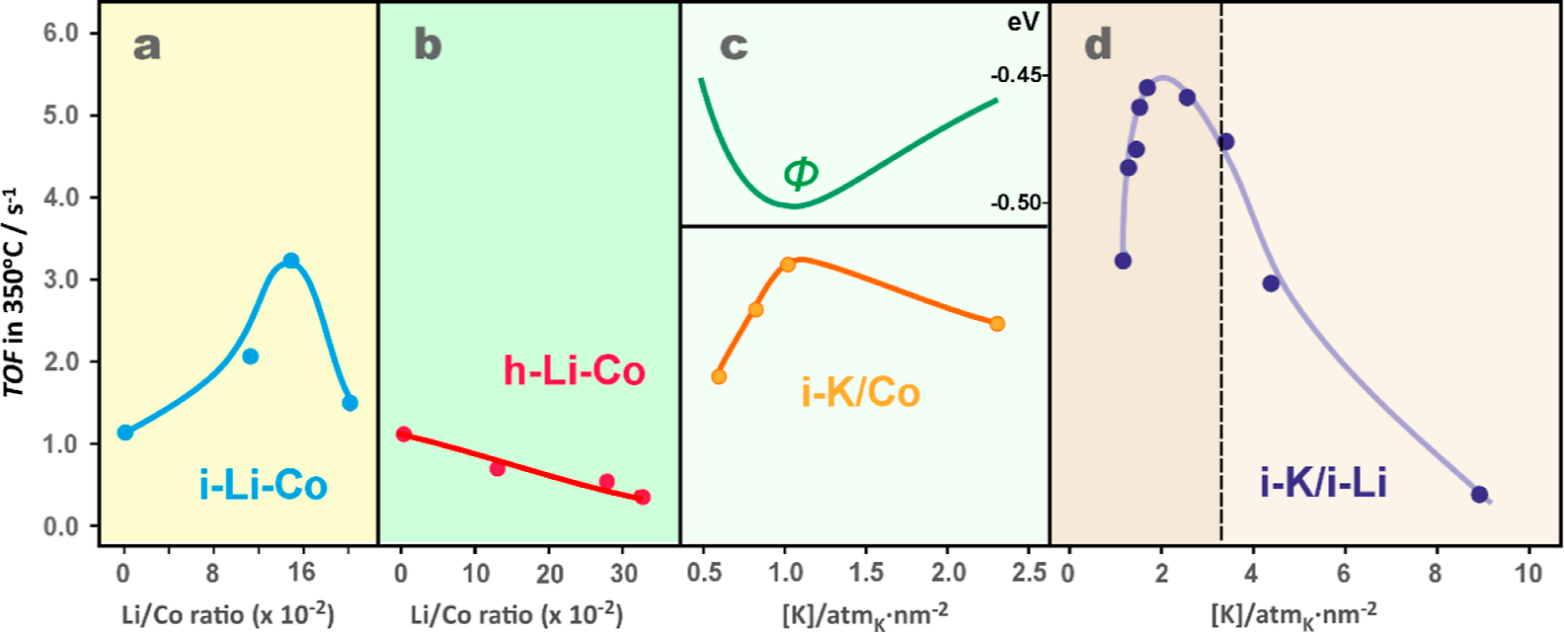
TOF values of the N_2_O decomposition at 350 °C for i-Li_*x*_Co_3_O_4_ (a), h-Li_*x*_Co_3–*x*_O_4_ (b), i-K_*y*_/Co_3_O_4_ (c), and i-K_*y*_/Li_*x*_Co_3_O_4_ (d) series of the cobalt spinel catalysts. The upper profile (marked green) in the panel (c) indicates the changes in the work function with surface potassium concentration (it represents a section of the complete plot of Φ, shown in Figure S9 in ESI).

The clear-cut nanocubic morphology of the investigated spinel catalysts (see Figure 1) allows for a reliable translation of the N_2_O conversion at the representative temperature of 350 °C into the turnover frequencies (eq 2). The resultant TOF values were plotted as a function of the doping level for the i-Li_*x*_Co_3_O_4_, h-Li_*x*_Co_3–*x*_O_4_, i-K_*y*_/Co_3_O_4_, and i-K_*y*_/Li_0.045_Co_3_O_4_ series (Figure 6a–d). The i-Li_*x*_Co_3_O_4_ catalysts are characterized by a distinct volcano-type dependence of TOF on the Li/Co ratio (Figure 6a), in contrast to the h-Li_*x*_Co_3–*x*_O_4_ series, where the TOF values are steadily decreasing in a rather flat way (Figure 6b).

The corresponding TOF profiles for the i-K_*y*_/Co_3_O_4_ and i-K_*y*_/Li_0.045_Co_3_O_4_ catalysts are plotted in Figure 6c,d. Additionally, the top panel in Figure 6c shows the changes in the work function, Φ, with the increasing surface potassium concentration. Like in the case of i-Li_*x*_Co_3_O_4_, the TOF values for i-K_*y*_/Co_3_O_4_ are also changing in a volcano fashion with increasing potassium loading. These changes are directly correlated with the work function in such a way that the lower the work function Φ (easier the electron transfer), the higher the activity (TOF value).

The performance of doubly promoted i-K_*y*_/Li_*x*_Co_3_O_4_ catalysts significantly outperforms those of the singly doped i-Li_*x*_Co_3_O_4_ and i-K_*y*_/Co_3_O_4_ counterparts, revealing a clear synergy of the lithium and potassium doping when both are introduced by the impregnation. The origin of this effect and the appearance of the TOF maximum are discussed in Chapter 3.4.

### 3.3 DFT Investigations into Structure-Redox Relationships

**Figure 7:**
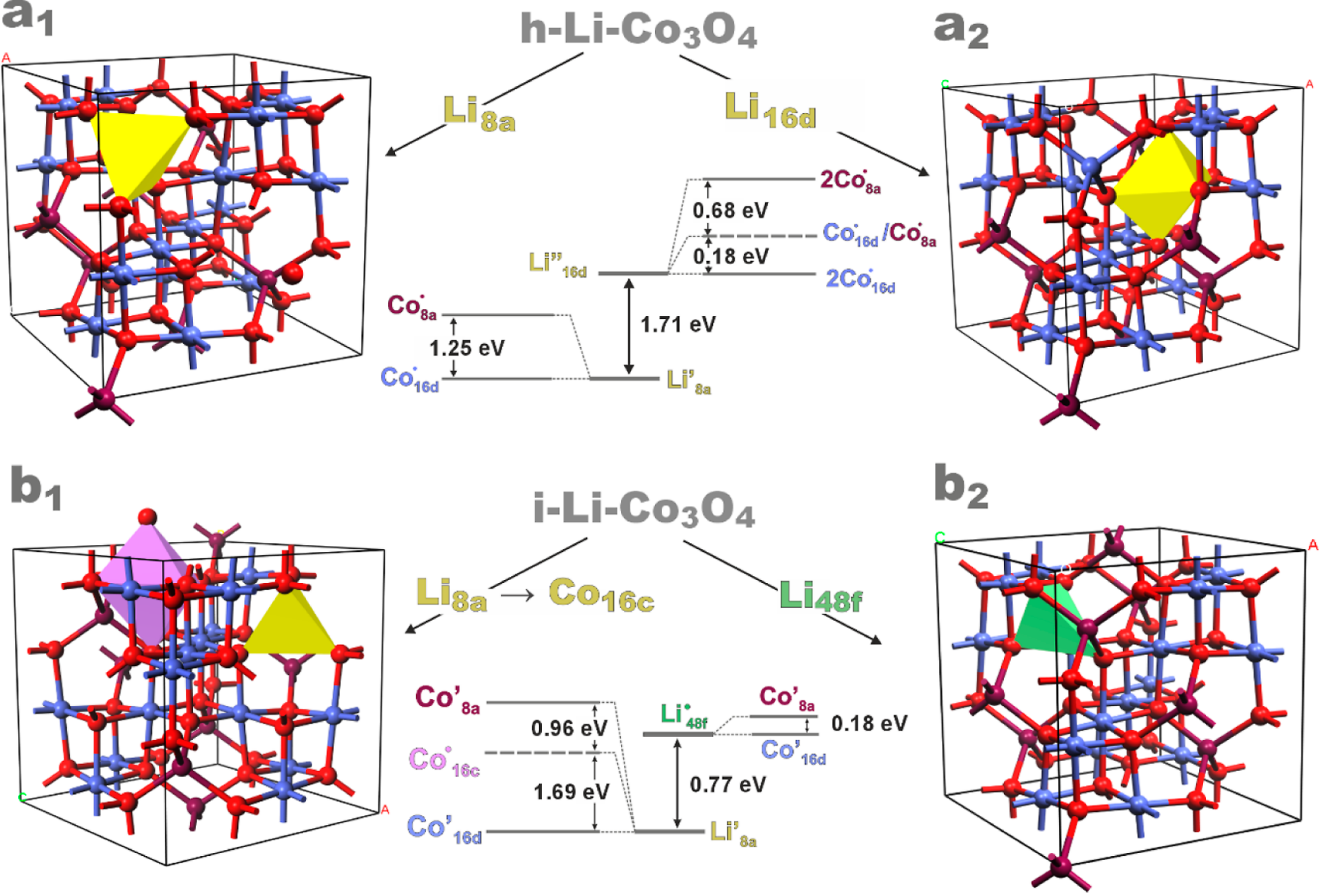
Location possibilities of the Li ions in the cobalt spinel host and the generated electron and hole-bearing defect centers in the case of the lithiated spinel catalyst obtained via one-pot synthesis (a_1_,a_2_) and by postsynthetic impregnation (b_1_,b_2_).

The optimized geometry of the parent bulk Co_3_O_4_ is shown in Figure S1, and the selected structural parameters are collated in Table S4. For modeling the incorporation of lithium into the cobalt spinel matrix, we examined four models of different lithium loci (Figure 7) to account for the two ways of Li doping, described in eqs 3 and 4a, 4b. In the case of the h–Li-Co_3_O_4_ catalysts, two possibilities of Li incorporation in the framework, tetrahedral or octahedral sites, were considered (see Figure 7a_1_,a_2_). The HSE06/DFT modeling confirmed the Li^+^ state of the lithium dopant, with the tetrahedral 8a (Li’_8a_) localization being more stable than the octahedral 16d (Li”_16d_) one, in accordance with the experimental findings.^85^ The corroborative OCC-MAT calculations indicated, in turn, that Li’_8a_ generates an electron hole, which is preferentially localized on the 16d cobalt, producing Co^4+^ (Co·_16d_) species. The formation of Co^4+^ is in accordance with the increased <AOS> value obtained from XAFS measurements (Figure 2) and the work function enhancement described above (Figure 3). The alternative location of the hole on an adjacent tetrahedral Co^2+^ cation (Co·_8a_) is disfavored by 1.25 eV. In the case of the less stable incorporation of Li in the octahedral sites, the created Li”_16d_ defect (Figure 7a_2_) is charge compensated by two holes, which are preferentially localized on the adjacent octahedral cations (formation of 2Co·_16d_ species). Other possible configurations, such as Co·_8a_/Co·_16d_ and 2Co·_8a_, are higher in energy (by 0.18 and 0.86 eV, respectively).

The two scenarios of Li incorporation through the impregnation method (i-Li_*x*_Co_3_O_4_ series) are illustrated in Figure 7b. The calculations reveal that the Li location in the framework 8a position (Li’_8a_, marked yellow in Figure 7b_1_) with the concomitant displacement of Co^2+^ from the 8a to 16c sites (Co··_16c_, marked in purple) is energetically more favorable than the location of Li at the interstitial 48f position (formation of Li·_48f_ species, marked green in Figure 7b_2_) by 0.77 and 0.95 eV for the charge-balancing electrons accommodated at the 16d (Co’_16d_) or 8a (Co’_8a_) sites, respectively. For the preferred Li’_8a_ center, the reduction of the octahedral framework Co^3+^ cations (Co’_16d_) is preferred over the interstitial octahedral Co^2+^ (Co·_16c_) and the framework tetrahedral Co^2+^ (Co’_8a_) cations by 1.69 and 2.65 eV, respectively (Figure 7b_1_).

The particular mechanisms of Li incorporation, ascertained by the OCC-MAT/DFT calculations, lead to a good agreement (within the error <5 ± 1%) between the experimental <AOS> values of cobalt derived from the XAFS plot (Figure 2d) and the <AOS> values calculated based on the established defect structure of the corresponding spinel catalysts [2.89 (XAFS) vs 2.72 (DFT) for h-Li_0.086_Co_2.94_O_4_ and 2.55 (XAFS) vs 2.65 (DFT) for i-Li_0.045_Co_3_O_4_]. This finding remains also in accordance with the observed and calculated work function changes as well (see Figure 3c,b).

In conclusion, the OCC-MAT/DFT computational results resolve the locus of Li dopants and the resulting valence pinning of the cobalt cations. In the case of the catalyst obtained via one-pot hydrothermal synthesis, the formation of pair {Li’_8a_, Co·_16d_} defects takes place upon the incorporation of lithium into the spinel matrix. The lithiation of spinel via postsynthesis impregnation leads, in turn, to the formation of triplet {Li’_8a_, Co’_16d,_ Co··_16c_} entities, upon the substitution of Li^+^ for Co^2+^. This is accompanied by the displacement of the tetrahedral Co^2+^ cations into the 16c interstitial octahedral sites.^86^

For the lithiated catalyst obtained by one-pot hydrothermal synthesis (h-Li_*x*_(Co_3-x_O_4_)), the formation of the {Li’_8a_, Co·_16d_} defects decreases the work function due to the *E*_F_ energy lowering, while for the Li-doped spinel obtained via postsynthesis impregnation, the formation of {Li’_8a_, Co’_16d,_ Co··_16c_} leads to an increase in the *E*_F_ level and the resultant lowering of the work function.

In the case of the (100)Co_3_O_4_ surface covered by the charged monatomic and diatomic oxygen intermediates, the observed increase in the ΔΦ value (Figure 3b,c) results from the formation of the surface dipoles, (ΔΦ_dip_ = *e* θ_ads_ μ_dip_/εε_0_), as already mentioned above. The drop in the ΔΦ value after potassium addition is caused by the inversion of the dipole moment (μ = 0.44 D) with respect to that produced by the anionic oxygen adspecies. The dipole moment resulting from the ionosorbed oxygen intermediates (calculated from the plane-averaged charge density difference along the *z*-direction^87^) is weaker (μ = 1.28 D) for the diatomic O_2_^–^ adspecies with the charge spread over both atoms, increasing significantly for the monatomic O^–^ intermediates (μ = 2.02 D). This explains properly the corresponding changes in the work function upon the temperature increase in terms of thermal dissociation of superoxide adspecies, which almost doubles the surface dipole moment. Notably, the sign and size of the surface dipoles produced are fully consistent with the observed and calculated work function changes (see Figure 3 and the associated discussion).

### 3.4 Conceptual Account of the Catalyst Redox Behavior

**Figure 8:**
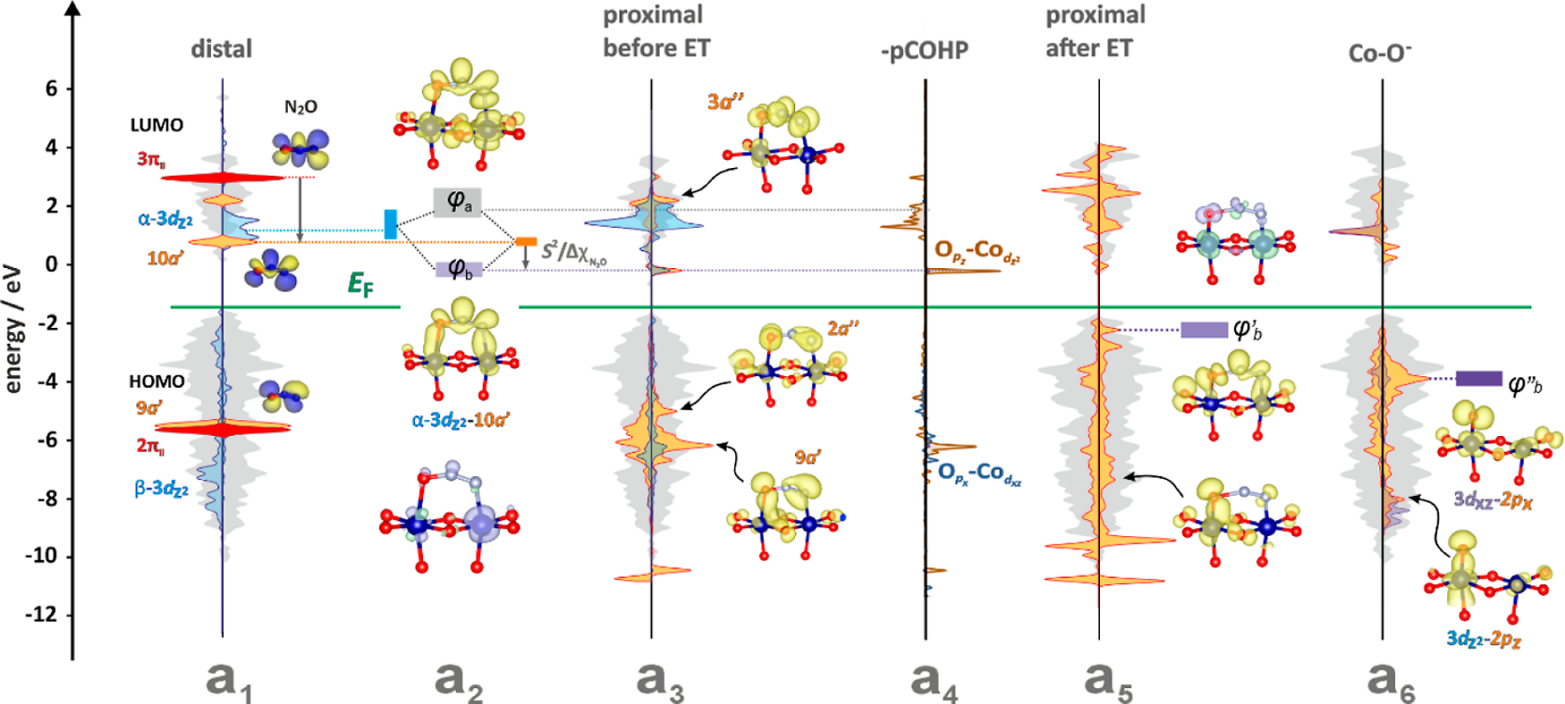
Molecular cascade of events in the course of N_2_O dissociation induced by an interfacial electron transfer, and the alignment of the energy levels of the prime molecular orbitals of N_2_O and the cobalt active centers. DOS structure of the (100) surface (gray shadowing) with the superimposed p-DOS of 3d_*z*2_ (marked in blue) and the FMO energy levels of the linear (marked in red) and bent (orange) N_2_O molecule in a distal configuration (a_1_), the key interaction diagram of the d_*z*2_-10*a*′ molecular orbitals before electron transfer (a_2_), constructed based on the DOS structure in a proximal configuration (a_3_), and the corresponding -pCOHP profile (a_4_). DOS structure in the proximal configuration upon electron transfer (a_5_), together with DOS of the resulting O^–^_ads_ intermediates produced upon N_2_O^–^ dissociation (a_6_). The spin density contours before (a_2_) and after electron transfer (a_5_) are colored pale green and pale violet, whereas the partial charge densities are colored pale yellow.

**Figure 9:**
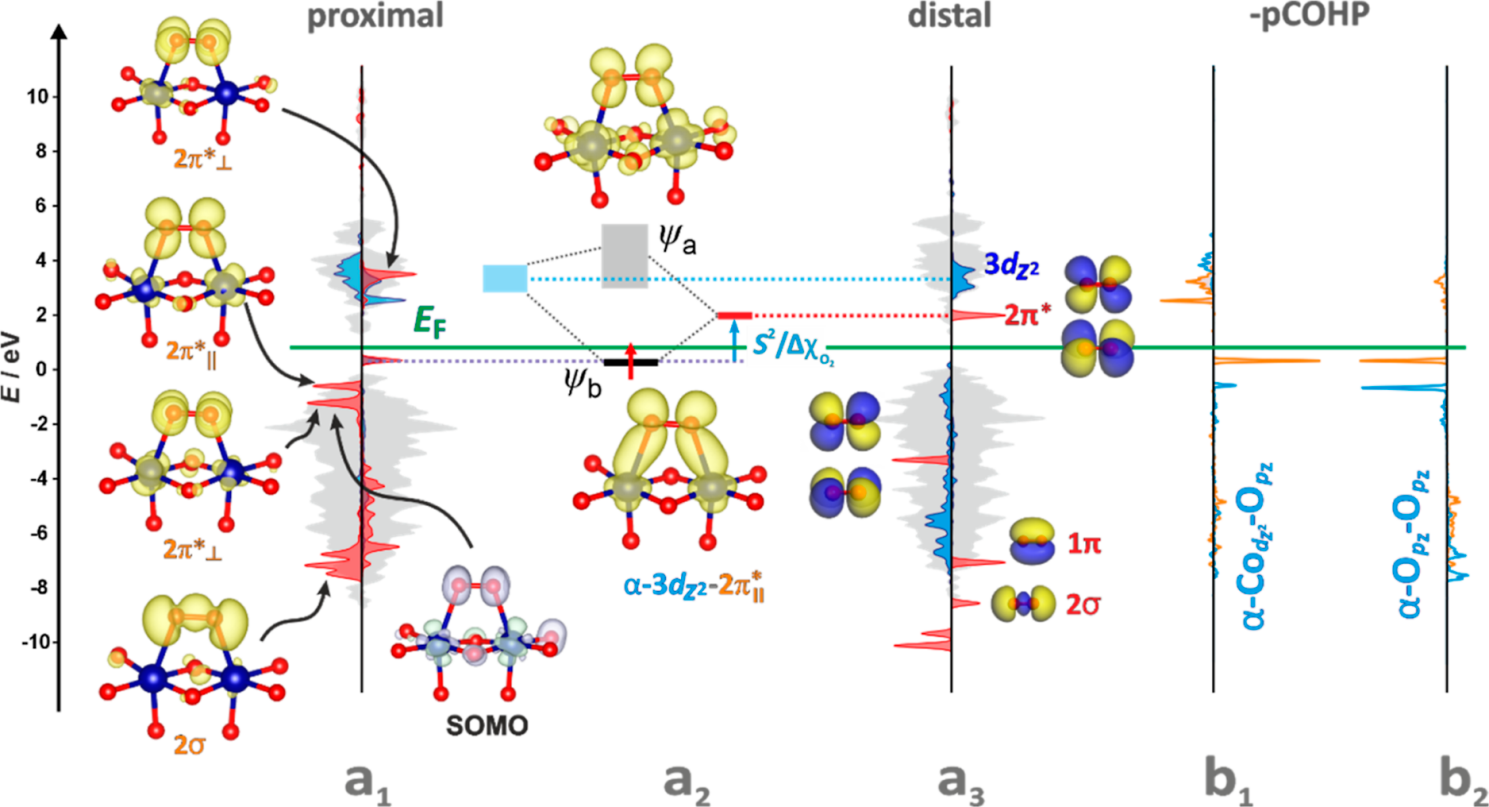
DOS structure of the (100) surface (marked by gray shadowing with the 3d_*z*2_ p-DOS component in marked blue) with the O_2_^–^ adspecies in the proximal configuration (marked in red) (a_1_). Molecular orbital interaction diagram for d_*z*2_-2π_||_*in the proximal configuration before (a_2_) and in the distal configuration after the back electron transfer (a_3_). The corresponding -pCOHP profiles are shown in (b_1_) and (b_2_), respectively. The spin density contour of SOMO (a_1_) is colored pale violet and green, whereas the partial charge densities are pale yellow.

The experimental results obtained, complemented by the calculated DOS structure of the spinel (100) surface and the energies of the FMO levels of N_2_O and O_2_, allowed us to construct a conceptual model of the redox interactions between the catalyst and the reacting molecules, build upon the tenets of molecular structure-based approaches to adsorption and catalysis.^41,43,48^ It provides a rational background for the elucidation of the effect of doping and charge buildup on the catalyst surface in the course of N_2_O decomposition. In the proposed treatment, the conceivable redox steps of nitrous oxide decomposition
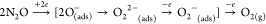
5are restricted to the two molecular events of an initial dissociative reduction of N_2_O (entrance into the catalytic cycle through an interfacial electron transfer, Figure 8), and a terminal evolution of O_2_ (exit from the catalytic cycle upon back electron transfer, Figure 9). The in-between molecular surface events of diffusive association of the O^–^_(ads)_ intermediates into transient O_2_^2–^_(ads)_, and their subsequent oxidative transformation into O_2_^–^_(ads)_ adspecies have been previously discussed by us,^61^ while a detailed molecular resolution of these surface steps will be elucidated in a forthcoming article.

Redox interactions at the N_2_O|(100)Co_3_O_4_ interface governing the catalyst activity depend critically on the surface DOS structure and the actual manifold of the FMO energy levels of the reactants and intermediates involved. To drive the N_2_O decomposition reaction, the surface cobalt active sites act as persistent interfacial electron shuttling centers and molecular templates, providing orbitals of proper energy and symmetry for N_2_O capture and efficient electron transfer. Because the neutral N_2_O is linear (C_∞v_), whereas the N_2_O^–^ transient is bent (C_s_), according to the Franck–Condon (FC) principle (the nuclear positions remain frozen during ET), the instantaneous geometries of both species must match to make the electron transfer feasible. This is associated with a significant internal reorganization energy of the N_2_O molecule [λ(N_2_O) = 2.3 eV]^88^, the distortion of which in a gas phase is used here as an explanatory example (see Figure S13). Upon the bending, resulting from the vibronic ν_2_ excitation (C_∞v_ → C_s_ transformation), the acceptor 3π* LUMO is significantly stabilized and becomes a 10*a*′ orbital (Figure S13a_1_). This is reflected in a remarkable enhancement in the electron affinity of N_2_O [from −2.1 (VEA) to = 0.22 eV (AEA)],^52^ which favors electron transference. The bending of N_2_O also leads to its inherent preactivation (Figure S13a_2_), manifested by a notable accumulation of the negative charge in the oxygen moiety (*q*_O_ = −0.2 |e|), and a convergence of the charge in both nitrogen atoms at the common value *q*_N_ = 0.1 |e|. These changes are accompanied by a substantial lengthening of the NN–O bond (by ∼0.3 Å), defining the actual dissociation coordinate of nitrous oxide. Once the electron is captured in the 10*a*′ orbital of the bent N_2_O molecule, the potential energy surface becomes dramatically flattened (cf. Figure S13b1,b2), making the resulting N_2_O^–^ transient species highly unstable. The FC locus for electron transfer is defined by the lowest energy crossing point between the N_2_O and N_2_O^–^ potential energy surfaces (see Figure S13b_3_, blue circle). Owing to the shallow minimum of the N_2_O^–^ potential energy surface, this transient species splits into N_2_ and O^–^ moieties spontaneously since the FC point is situated well above the N_2_O^–^ dissociation barrier. This means that the NN–O bond-breaking process is inherently induced by ET, and a significant part of the activation energy for the N–O bond-breaking results from the geometrical reorganization of the N_2_O molecule [λ(N_2_O)], which is imposed by the Franck–Condon constraint.

As reported by us, the sustainable active sites for the decomposition of N_2_O over the Co_3_O_4_ are constituted by the octahedral cobalt (Co_16d_) cations.^35^ The DOS structure of the surface (100) (see Figure S14) shows that the pentacoordinate Co^3+^ active sites (C_4v_) assume an open shell (d_*xy*_^2^, d_*yz*_^2^, d_*xz*_^1^, d_*z*2_^1^) configuration. In the case of the d_*z*2_ orbitals protruding toward the reactants, a strong electron exchange locates the empty α-d_*z*2_ state 1.8 eV above the *E*_F_ level, whereas the occupied β-d_*z*2_ counterpart situated below *E*_F_ is significantly broadened, ranging from −0.5 to −8 eV, with the maximum around −6 eV. For N_2_O reduction, the requisite electron has to be accommodated in LUMO (3π*), which is located too high in energy with respect to the Fermi level to make ET thermodynamically feasible. The corresponding lineup of the (100) surface DOS with the frontier orbitals of the N_2_O reactant is shown in Figure 8.

In the distal configuration, the HOMO (2π) level of N_2_O is deeply (−6 eV) plunged in the spinel valence band (VB), whereas the antibonding LUMO (3π*) is situated in the conduction band (Figure 8a_1,_ see DOS features marked in red). Both are then redox inactive, and since *E*(LUMO) ≫ *E*_F_ prevents the electron transfer to occur, the N_2_O molecule remains intact. However, vibrational excitation (ν_2_ = 596.3 cm^–1^) leads to N_2_O bending (3π* → 10*a*′ transformation), which strongly stabilizes the energy of the in-plane LUMO (see DOS features marked in yellow), as discussed above. Within the constraints imposed by the MO symmetry and energy matching rules, the 10*a*′ orbital of the bent N_2_O molecule can overlap most efficiently with the close-lying empty α-d_*z*2_ orbital of the cobalt active site (marked in blue). Following the Hoffman treatment of bonding on surfaces^89^ in a proximal configuration, such interaction leads to the formation of virtual bonding (ϕ_b_ = 10*a*’ + d_*z*2_) and antibonding (ϕ_a_ = d_*z*2_ – 10*a*′) states. The resultant molecular orbital diagram is shown in Figure 8a_2_, where the positions and energy dispersion of the ϕ_b_ and ϕ_a_ levels were determined from the calculated DOS structure of the N_2_O|Co_3_O_4_ system in the proximal configuration (Figure 8a_3_). Their bonding and antibonding character was ascertained by using the corresponding pCOHP diagram (Figure 8a_4_). Hitherto, a small interaction between the occupied β-d_*z*2_ and β-10*a*′ states contributes to an incipient association of the N_2_O molecule, similarly to the weaker out-of-plane overlap between the 3*a*” and 3d_*yz*_ orbitals, but these interactions were neglected for the sake of simplicity. The extent of ϕ_a_ stabilization is proportional to the *S*^2^/Δχ_N_2_O_ term (where *S* is the overlap integral and Δχ_N_2_O_ is the energy difference between the parent d_*z*2_ and 10*a*′ levels in the distal position). The ϕ_b_ state, although initially empty (see the corresponding spin density contour in Figure 8a_2_ with the spurious amount of ρ_spin_ on the N_2_O moiety only), plays a critical role in N_2_O activation. The position of this energy level relative to the Fermi energy gauges for the amount of energy needed to trigger the interfacial electron transfer. An increasing hybridization (∼*S*^2^/Δχ_N_2_O_) between the 10*a*′(N_2_O) and 3d_*z*2_ Co orbitals, induced by shrinking the Co-ON_2_ distance, can drive the virtual ϕ_b_ below *E*_F_ (at d(Co-ON_2_) ∼ 1.7 Å), making ET thermodynamically feasible. This is accompanied by dramatic changes in the hybridization of the resultant (ϕ′_b_)^1^ orbital, caused by the lengthening of the O–NN bond (from 1.26 to 1.67 Å), and incipient severance of the dinitrogen molecule (see the developing in-plane π_||_ and out-of-plane π_⊥_ orbitals of the nascent N_2_ in Figure 8a_5_). At this stage of the reaction, the ϕ′_b_ state, apart from the dominant parent 2p_*z*_ (0.38) and 3d_*z*2_ (0.1) contributions, also acquires considerable 2p_*x*_(0.14) and 3d_*xz*_ (0.04) character. Such dramatic changes in the molecular structure of the N_2_O moiety, caused by the electron capture, are reflected in a pronounced enhancement of the total IpCOHP value of the Co–N_2_O–Co bond from −3.6 to −9.1 eV. The neutral N_2_O molecule is more strongly attached via the N atom (IpCOHP = −2.28 eV) than the O atom (IpCOHP = −1.33 eV), whereas in the case of the N_2_O^–^ ligand, the O–Co bond becomes slightly stronger than the N–Co one (−4.84 eV vs −4.29 eV). In particular, the interfacial electron transfer results in a spectacular enhancement of the IpCOHP value for the key α-d_*z*2_-2p_*z*_(O–Co) interaction realizing this process, which increases from −0.26 to −1.99 eV. The lifetime of the N_2_O^–^_(ads)_ transient, τ(N_2_O^–^) ∼ 400 fs, estimated from the hybridization function of LUMO using the Newns-Anderson model,^72^ is sufficiently large in comparison to the NN–O stretching time,τ_N–O_ ∼ 5 fs, which triggers successful dissociation of the N_2_O^–^ molecule. Since within this approach, the N_2_O^–^ lifetime is determined by the broadening of the (ϕ′_b_)^1^ state due to the local interactions with the 3d orbitals of the cobalt adsorption site, it is not directly sensitive to the distal/neighboring dopants. Notably, the estimated value of τ(N_2_O^–^) is of the same order in magnitude as that reported for N_2_O^–^ trapped on a titania catalyst (100 fs).^50^

The corresponding spin density plot (Figure 8a_5_) confirms the pronounced accumulation of the unpaired electron on the oxygen atom of the N_2_O^–^ transient, consistent with the SOMO character of ϕ′_b_. Upon final detachment of the N_2_ moiety, ϕ′_b_ evolves into the occupied p_*z*_(O)–d_*z*2_ and p_*x*_(O)–d_*xz*_ (ϕ”_b_) states, revealing a strong electronic relaxation of the redox core once the electron transfer process is fully accomplished. After N_2_ release_,_ the unpaired electron is shifted into the ϕ”_b_ orbital of the ensuing O^–^ adspecies (Figure 8a_6_), while the p_*z*_(O)–d_*z*2_ part of the original 10*a*’–3d_*z*2_ interaction, left after N_2_O dissociation, becomes deeply immersed in the VB band.

As mentioned above (see eq 5), the evolution of the final O_2(g)_ is initiated by the recombination of the two O^–^_(ads)_ intermediates into a transient O_2_^2–^_(ads)_ adspecies, which is next oxidized into O_2(g)_ by a gradual release of the 2 electrons back to the cobalt active sites (closing the redox cycle). The orbital interaction diagram for the last step of this process, O_2_^–^_(ads)_ O_2(g)_, is shown in Figure 9, where the key role is played by the singly occupied bonding orbital, ψ_b_, that is located just below the *E*_F_ level, and resulting from an overlap between the α-2π_||_* orbital of the superoxide intermediate and the α-3d_*z*2_ orbital of cobalt (see Figure 9a_1_,a_2_).

The bonding character between Co–O_2_^–^, and the antibonding character between O–O is ascertained by analysis of the pCOHP profiles shown in Figure 9b_1_,b_2_, respectively. Since the ψ_b_ level is located below *E*_F_, the back electron transfer can be achieved by decreasing the overlap term (*S*^2^/Δχ_O2_), realized simply by lengthening the Co–O_2_^–^ distance above 2.4 Å. The mutual DOS-MO alignment of the released O_2_ molecule (in distal configuration) and the α-3d_*z*2_ state of the restored cobalt active center is shown in Figure 9a_3_. The orthogonal β-2π_⊥_* overlapping with β-3d_*xz*_ corresponds to SOMO (see Figure 9a_1_, and the corresponding spin density spatial distribution). Thus, the electronic configuration of the bound O_2_^–^ moiety can be formulated as (β-2π_⊥_*)^1^(β-2π_||_*)^1^(α-2π_||_*)^1^(α-2π_⊥_*)^0^. As a result, the spin-polarized back electron transfer (removal of an electron from α-2π_||_*) leads directly to dioxygen release in its triplet ground state ^3^Σ^–^_g_.

**Figure 10:**
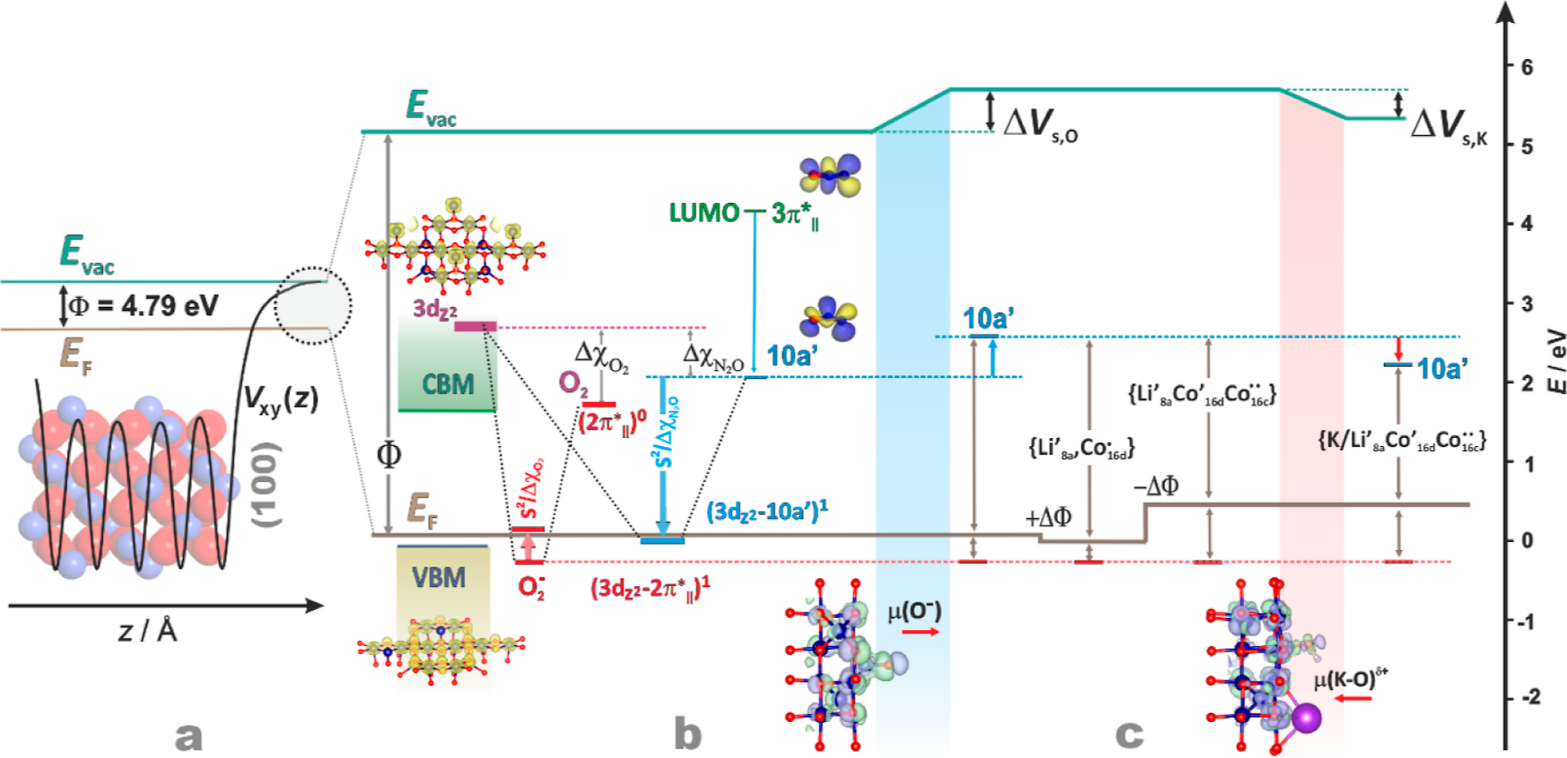
Conceptual redox diagram for N_2_O decomposition on cobalt spinel catalysts. Plane-averaged electrostatic potential profile, *V*_*xy*_(*z*), defining the local vacuum level (*E*_vac_), the work function for the bare Co_3_O_4_ (a), the surface DOS structure with the energy levels of the redox orbitals of N_2_O, O_2_, and O_2_^–^ (b), changes in the surface potential and the Fermi level, caused by the accumulation of charged oxygen intermediates on the surface and the doping of spinel by Li and K (c).

The conceptual diagram for the redox interaction of a N_2_O molecule with the (100) surface of the parent, singly, and doubly doped Co_3_O_4_ is shown in Figure 10. For the determination of the local vacuum level (*E*_vac_) for the bare and covered (100) surface of Co_3_O_4_, we calculated the plane-averaged electrostatic potential profiles along the ^100^ direction, *V*_*xy*_(*z*).^87^ The distance between the Fermi level (*E*_F_) and *E*_vac_, shown in Figure 10a, corresponds to the work function for the bare surface, Φ_DFT_ = 4.8 eV, which agrees quite well with the experimental value of 4.98 eV (Figure 3c). The alignment of the LUMO levels for the linear (3π*_||_) and bent (10*a*′) N_2_O reactant, and the SOMO of the O_2_ product (2π*) with respect to the valence and conduction bands of the (100) Co_3_O_4_ surface (their composition is represented by the 3D partial charge densities, averaged in the range of 0÷0.5 eV below VBM and above the CBM), is shown in Figure 10b. At distal configuration, the MO energy levels of N_2_O_(g)_ and O_2(g)_ are referenced to a common local *E*_vac_ level. The distance of LUMO (N_2_O) and SOMO (O_2_^–^) to the catalyst Fermi energy position controls the feasibility of forth/back electron transfer that turns the catalytic cycle. According to the Fermi–Dirac distribution, it is also influenced by the temperature of the reaction. As a result, the *E*_F_ energy, which is equivalent to the chemical potential of the electrons in the spinel catalyst, plays a key threshold function in these processes.

As explained above, the LUMO level of N_2_O is lowered upon bending and hybridization with the cobalt 3d_*z*2_ orbital (∼*S*^2^/Δχ_N_2_O_) to reach the threshold level of *E*_F_, where ET can occur spontaneously. Above *E*_F_, the electron transfer may arise as a thermally activated process only. Thus, an apparent Δ*E*_a_ value depends, *i*.*a.*, on the amount of the 3d_*z*2_–10*a*′ overlap, controlled by the N_2_O approach to the surface cobalt active sites, and the reorganization energy needed for N_2_O bending [λ(N_2_O)].

For the evolution of dioxygen, which closes the catalytic cycle, the (3d_*z*2_-2π*_||_) level must be elevated above *E*_F_ to transform the O_2_^–^_(ads)_ intermediates into the O_2(gas)_ molecules via back electron transfer. This can be achieved by lowering the hybridization extent with the catalyst surface (*S*^2^/Δχ_O2_), simply by increasing the Co–O_2_ distance above 1.6 Å. The uneven gap between the 3d_*z*2_-10*a*’ (blue arrow) and 3d_*z*2_-2π*_||_ levels (orange arrow) and the Fermi energy implies that the reduction of N_2_O should energetically be more demanding than the oxidation of the O_2_^–^_(ads)_ intermediate into the final dioxygen product, in a straight accordance with the apparent reaction order close to one (see Figure S12c and the associated text in the ESI Section).

The described picture corresponds to N_2_O decomposition for *p*_N_2_O_ → 0, where the surface coverage by the charged oxygen intermediates (O^–^ and O_2_^–^) is negligible. In the case of *p*_N_2_O_ = 50 hPa, the observed increase of the activation energy by 0.5 eV (from Δ*E*_a_ = 0.66 to 1.16 eV, see Section 3.2) is associated with the buildup of the surface potential Δ*V*_s,O_ ≈ 0.49 eV (reflected by an increase of the work function value) (ΔΦ_dip_ = Δ*V*_s,O_) due to partial coverage of the surface by the anionic oxygen intermediates. The resultant shift of the local *E*_vac_ level moves the 10*a*′ energy away from *E*_F_ (marked by the blue arrow in Figure 10c), making the interfacial electron transfer more demanding. This leads to the apposite augmentation of the activation energy for N_2_O dissociation by 0.5 eV, as experimentally observed.

The key role of the Fermi level in the redox behavior of the spinel catalyst is further substantiated by taking into account the electronic effects of the Li and K doping, described in Sections 3.1 and 3.3. In the case of the h-Li_*x*_Co_3-x_O_4_ series obtained by hydrothermal synthesis, the generated {Li’_8a_, Co·_16d_} species (defects) lower the *E*_F_ level (Φ increases). This augments the barrier for the ET, in accordance with the observed lower activity of these catalysts, which steadily varies in parallel with Φ (see TOF values in Figure 6b and *E*_a_ in Figure S11).

The opposite effect is observed for the i-Li_*x*_Co_3_O_4_ series obtained by the impregnation, which leads to the generation of the {Li’_8a_, Co’_16d,_ Co··_16c_} entities. The latter raises the *E*_F_ level, decreasing the barrier of the fourth electron transfer, which is reflected in the significant enhancement of the TOF values in comparison to the bare spinel (see Figure 6a). However, the catalytic activity pattern is actually more involved, passing through the maximum with increasing Li/Co ratio. This phenomenon can be accounted for by a trade-off between two opposite effects. Enhancement of the *E*_F_ level increases the deN_2_O activity, which favors the accumulation of the ionosorbed oxygen intermediates (by shifting the reaction to lower temperatures where the oxygen adspecies are more stable). However, the latter effect by increasing the Δ*V*_S,O_ value hinders the ET activation of N_2_O, lowering the TOF values for higher Li/Co ratios (see Figure 6a). Furthermore, the presence of the interstitial cobalt (Co··_16c_) cations increases the energy of the dioxygen desorption from 0.65 to 0.91 eV for bare Co_3_O_4_ (Co_16d_-O_2_-Co_16d_) and i-Li_*x*_Co_3_O_4_ (Co_16d_-O_2_-Co_16c_) catalysts, respectively (see Figure S15), favoring thereby an accumulation of the charged oxygen intermediates on the catalyst surface.

The resultant surface potential buildup of the oxygen intermediates can be mitigated by the addition of potassium. The created surface dipole (μ = 0.44 D) due to potassium adspecies is of the opposite direction to that produced by the anionic oxygen intermediates (μ = 1.28 and 2.02 D for O_2_^–^ and O^–^, respectively), lowering the energy separation between the 10*a*′ LUMO level of N_2_O and *E*_F_ (marked by the red arrow), see Figure 10c. Within this account, the nonmonotonous changes of the TOF values for the i-K_*y*_/Co_3_O_4_ catalyst (Figure 6c) result from the parallel variation of the work function upon doping (see the green profile in the upper panel).

The volcano dependence on K loading is repeated in the case of the doubly doped i-K_*y*_/Li_*x*_Co_3_O_4_ catalysts of the top activity (see Figure 6d). The appearance of the TOF maximum can be traced back to the synergy between the beneficial enhancement of the *E*_F_ level (Li doping) and the lowering of the surface potential built-up (K-doping). The left part in Figure 6d corresponds to the work function changes controlled by the Topping model, in full analogy to the singly K-doped spinel (Figure 6c). Yet, excessive loading of potassium leads to an apparent steric blocking of surface active sites, and the TOF values drop eventually below the value observed for the unpromoted spinel (see Figure 6d right part).

## 4 Conclusions

The redox properties of cobalt spinel can be tuned on purpose by aliovalent doping with Li ions. The one-pot hydrothermal lithiation leads to the generation of {Li’_8a_, Co·_16d_} species (defects) that lower the *E*_F_ level and the deN_2_O activity in a commensurate way. Postsynthetic lithiation via impregnation leads to the generation of the {Li’_8a_, Co’_16d_, Co··_16c_} species and volcano-type dependence of the TOF values on the Li content. The key factors governing redox activity of the cobalt spinel catalyst include: lineup of the MO energy levels of the reactants and the surface DOS structure, reorganization energy associated with the N_2_O bending to satisfy the Franck–Condon constraint, strength of the mutual interactions of the LUMO (N_2_O) and SOMO (O_2_^–^_ads_) with the 3d_*z*2_ (Co) orbitals, and buildup of surface potential due to accumulation of anionic oxygen intermediates (surface electrostatics). The established molecular orbital pattern and the position of the Fermi level control the trade-off between the interfacial electron transfer that triggers N_2_O dissociation and the back electron transfer that drives the subsequent dioxygen evolution. The crucial role of overlap between the virtual 10*a*′-3d_*z*2_ orbitals in attaining the transference of electrons between the N_2_O reactant (acceptor center) and the octahedral cobalt active sites (donor center) was shown for the first time.

The constructed conceptual framework allows for a concise account of the multifaceted redox features of the N_2_O decomposition reaction on cobalt spinels. It extends the classic picture of catalysis on semiconductors, pioneered by Wolkenstein and Hauffe, primarily by including a key hybridization of the molecular orbitals of the reacting species with the catalysts DOS features, surface electrostatics, and the reorganization energy of reactants. The proposed model of redox behavior can be applied to any *p*-type semiconductor catalysts for a straightforward rationalization of the molecular structure-redox reactivity relationships.

## Supporting Information Available

Structure of Co_3_O_4_, slab models for cobalt spinel molecular modeling, characterization of the catalysts, catalytic performance, basic structural, and electronic characterization of Co_3_O_4_, Walsh diagram and potential energy surfaces of N_2_O and N_2_O^–^, DOS structure of the (100) surface of Co_3_O_4_, pDOS profiles of the exposed truncated octahedral Co^oct^_5C_ cations, and energetic profiles for oxygen evolution on bare and Li-doped Co_3_O_4_ (PDF)
