# Supplementary material for: N2O Decomposition on Singly and Doubly (K and Li)-Doped Co3O4 Nanocubes—Establishing Key Factors Governing Redox Behavior of Catalysts
Source: J Am Chem Soc. 2024 Aug 23;146(35):24450–66. doi: 10.1021/jacs.4c06587 (PMC11378300; doi:10.1021/jacs.4c06587)
Supplement: Supplementary file 1 — ja4c06587_si_001.pdf [file ja4c06587_si_001.pdf]

Supporting Information for publication:

N_2O Decomposition on Singly and Doubly (K and Li) Doped Co_3O_4 Nanocubes – Establishing Key Factors Governing Redox Behavior of the Catalysts

Leszek Nowakowski[a,d], Camillo Hudy[a], Filip Zasada[a], Joanna Gryboś[a], Witold Piskorz[a], Anna Wach[b], Yves Kayser[c], Jakub Szlachetko[b], Zbigniew Sojka[a,*]

[a]*Faculty of Chemistry Jagiellonian University, ul. Gronostajowa 2, 30-387 Krakow, Poland*

[b]*National Synchrotron Radiation Centre SOLARIS Jagiellonian University ul. Czerwone Maki 98, 30-392 Kraków, Poland*

[c]*Physikalisch-Technische Bundesanstalt (PTB), Abbestr. 2-12, 10587 Berlin, Germany*
Current affiliation: Max Planck Insitute for Chemical Energy Conversion, Stiftstr. 34-36, 45470 Mülheim an der Ruhr, Germany

[d]*Doctoral School of Exact and Natural Sciences, Jagiellonian University, Prof. St. Łojasiewicza St 11, 30-348, Krakow, Poland*

**Corresponding author: e-mail: sojka@chemia.uj.edu.pl*

Chapter S1. Structure of Co_3O_4

The cobalt spinel oxide (Co_3O_4) structure, belonging to the *Fd*3*m* symmetry group, features a unit cell with 8 formula units ($Co_{24}O_{36}$ stoichiometry) and cell constant *a* of 8.149 Å, as illustrated in **Figure S1a**. In this structure, the oxygen anions (shown in red) occupy the 32e Wyckoff positions, creating an FCC lattice. In this lattice, one-eighth of the available tetrahedral sites (8a positions) are occupied by divalent cations (purple), while half of the octahedral sites (16d positions) are occupied by trivalent cations (blue), consistent with a normal spinel structure. The unoccupied tetrahedral (48f) and octahedral (16c) sites are depicted by green and violet polyhedra, respectively.

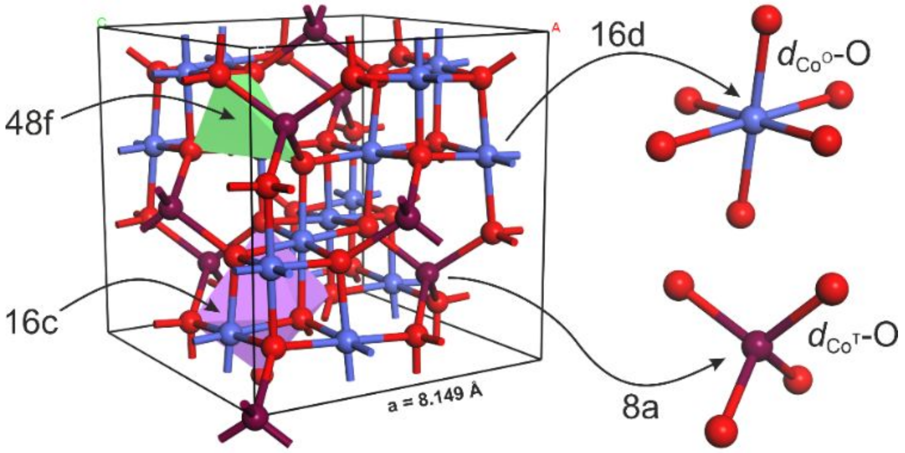

Figure S1. Co_3O_4 unit-cell revealing most important cationic sites, and empty positions within FCC oxygen lattice.

Chapter S2. Slab models for cobalt spinel molecular modeling

To compare experimental and theoretical work function values slab models exposing the (100) surface of the spinel catalyst, differing in K and Li doping and oxygen coverage, were developed. The side view of the slab model for the bare (c-Co_3O_4) cobalt spinel is shown in **Figure S2a$_1$**, whereas the perspective view of its topmost atomic layer is shown in **Figure S2a$_2$**. The exposed surface element is composed of tetrahedral (Co_{8a}) and octahedral (Co_{16d}) cations, as well as tri- and tetra-coordinated oxygen anions.

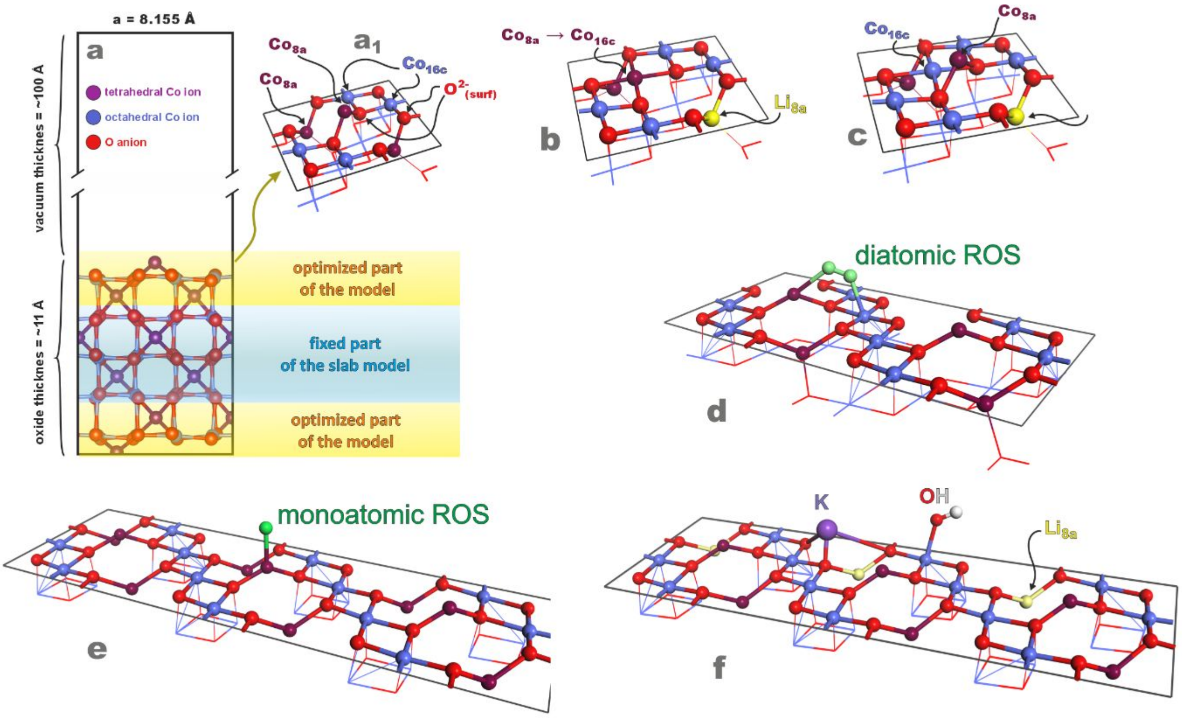

Figure S2. Slab models for WF calculations of cobalt spinel catalysts. Bare cobalt spinel (**a**), lithium-doped catalyst obtained via one-pot synthesis (**b**), and via impregnation (**c**), catalyst covered with diatomic (**d**) and monoatomic (**e**) oxygen species, and catalyst decorated with potassium (**f**).

Figure S2b shows the catalyst's surface doped with lithium via one-pot hydrothermal synthesis, where the Li cations are directly incorporated in the spinel matrix. Li preferentially replaces cobalt in its 8a positions (see **Figure 7** in the main text), forming $Li_{(8a)}$ species, which shifts the exposed tetrahedral cation to an adjacent empty vacancy ($Co_{8a} \rightarrow Co_{16c}$). In the case of the Li-impregnated

catalyst (**Figure S2c**), the substitution of Li^+ for the tetrahedral $Co^{2+}{}_{8a}$ cation is expected, and the latter is shifted from the framework 8a into the interstitial octahedral 16c position. The models representing spinel surfaces covered with diatomic (O_2^- species stabilized on the Co_{16c}‿Co_{16d} cationic pair, **Figure S2d**) and monoatomic oxygen adspecies (the most stable Co_{8a}-O metal-oxo surface adducts, **Figure S2e**) were based on our previous work [1]. In both cases, to account for the surface coverage the slab unit cell was enlarged by 2×1 and 3×1, respectively (see **Figures S2e** and **S2d**). The potassium doping was modeled as KOH adspecies [2] using the 3×1 slab (see **Figure S2f**).

The work function, Φ, was calculated based on the electronic structure of the developed slab models as the difference between the Fermi level (E_F) and a sum of the barrier ($E_{barrier}$) and vacuum energy (E_{vacuum}) of the plane plane-averaged outer electrostatic potential (for details see **Figure S3a** and **S3b**). The dipolar contribution (generated by the oxygen adspecies and the surface potassium dopant) was included when needed.

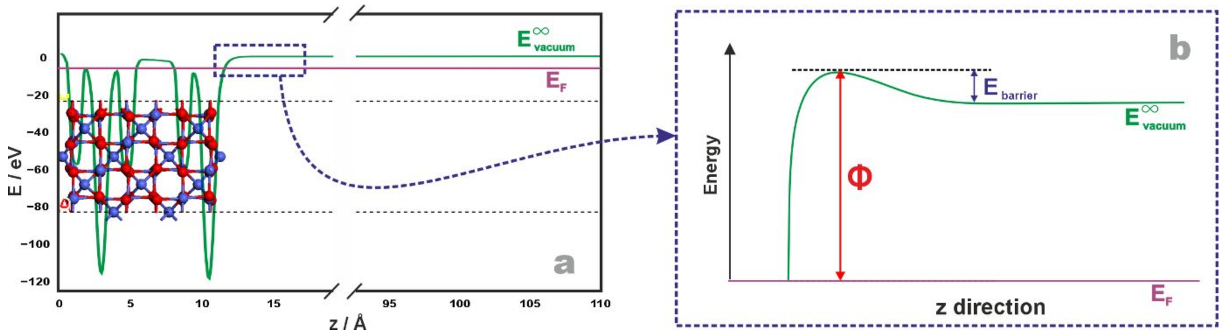

Figure S3. The electron potential energy diagram above a cobalt spinel (100) surface with Fermi level and vacuum level indicated (**a**) together with an enlarged near-surface area showing the energy barrier for electron extraction (**b**).

Chapter S3. Characterization of the Catalysts

In contrast to potassium, Li is readily incorporated into the spinel matrix, and the deficient atomic sensitivity factor of Li (0.02) precludes assessment of its actual surface concentration by XPS quantification, as the corresponding signals were not resolved above the background noise level. Therefore, the surface concentration of Li could not be reliably determined thus only the Li/Co ratio is reported.

Table S1: Composition of the auxiliary broad range series of lithiated cobalt spinel catalyst expressed as Li/Co ratio. For the labeling of the series, we used ’ mark to distinguish it from the prime narrow range series of the catalysts.

Catalyst labeling	[Li]/[Co] ratio
Li-1’-Co	3.29×10^{-3}
Li-2’-Co	2.77×10^{-2}
Li-3’-Co	3.69×10^{-2}
Li-4’-Co	4.61×10^{-2}
Li-5’-Co	9.23×10^{-2}
Li-6’-Co	1.85×10^{-1}
Li-7’-Co	2.31×10^{-1}

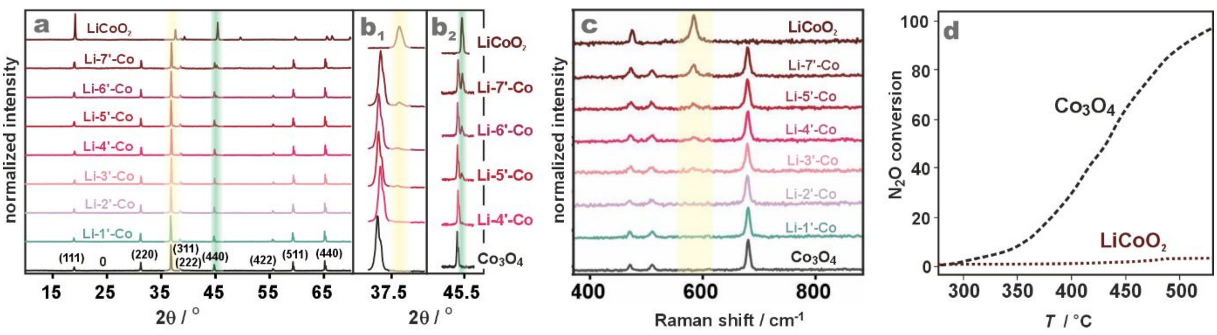

Figure S4. XRD patterns of the broad range auxiliary series of the lithiated cobalt spinel catalyst (**a**) with enlarged fragments around 37.5° (**b$_1$**) and 45.5° (**b$_2$**) 2Θ values, along with the corresponding Raman spectra recorded with the 514 nm laser (**c**) and N_2O conversion comparison between Co_3O_4 and $LiCoO_2$ (**d**).

Table S2. Composition of prime series of the Li-doped Co_3O_4 catalysts with Li/Co ratio determined by ICP-MS. The i-$Li_xCo_3O_4$ series was obtained by Li introduced via impregnation of the cobalt spinel nanocubes, whereas the h-$Li_xCo_{3-x}O_4$ series was obtained via one-pot hydrothermal method.

Catalysts	Catalyst labeling	Li/Co ratio actual	x value
i-$Li_xCo_3O_4$	i-Li-1-Co	0.011	0.033
	i-Li-2-Co	0.015	0.045
	i-Li-3-Co	0.020	0.060
h-$Li_xCo_{3-x}O_4$	h-Li-1-Co	0.013	0.040
	h-Li-2-Co	0.028	0.086
	h-Li-3-Co	0.032	0.099

Table S3. Composition of the i-K_y/Co_3O_4 and i-$K_y/Li_{0.045}Co_3O_4$ (i-K_y/Co-Li-2) catalysts series (potassium content determined by XRF), along with the surface concentration of potassium expressed as a number of K atoms per square nanometer, using the surface area values determined by BET measurements. The actual concentration of potassium is close to the nominal one as seen in the inserted figure.

catalyst	Catalyst labeling	[K]/[Co] ratio	*y value*	$n_K/atm_K\cdot nm^{-2}$
i–K_y/Co_3O_4	i-K-1/Co	4.02×10^{-4}	1.21×10^{-03}	0.60
	i-K-2/Co	5.55×10^{-4}	1.67×10^{-03}	0.84
	i-K-3/Co	6.71×10^{-4}	2.01×10^{-03}	1.01
	i-K-4/Co	1.53×10^{-3}	4.59×10^{-03}	2.31
i-$K_y/Li_{0.045}Co_3O_4$	i-K-1/i-Li-2-Co	4.31×10^{-4}	1.29×10^{-03}	0.65
	i-K-2/i-Li-2-Co	4.74×10^{-4}	1.42×10^{-03}	0.72
	i-K-3/i-Li-2-Co	5.82×10^{-4}	1.75×10^{-03}	0.88
	i-K-4/i-Li-2-Co	6.25×10^{-4}	1.88×10^{-03}	0.94
	i-K-5/i-Li-2-Co	6.92×10^{-4}	2.08×10^{-03}	1.05
	i-K-6/i-Li-2-Co	1.32×10^{-3}	3.96×10^{-03}	1.99
	i-K-7/i-Li-2-Co	1.88×10^{-3}	5.64×10^{-03}	2.84
	i-K-8/i-Li-2-Co	2.53×10^{-3}	7.59×10^{-03}	3.82
	i-K-9/i-Li-2-Co	5.52×10^{-3}	1.66×10^{-02}	8.34

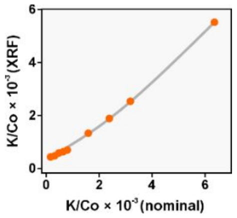

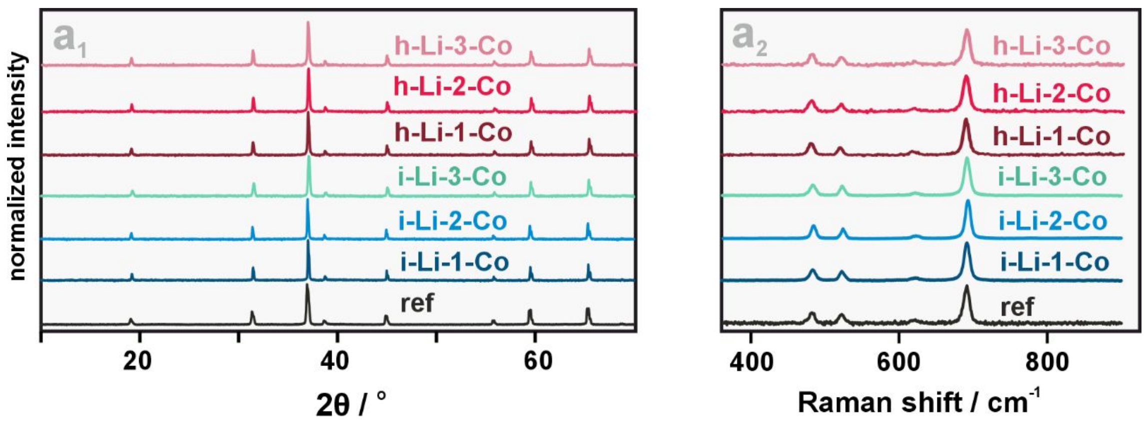

Figure S5. Powder X-ray diffraction patterns (**a**), and Raman spectra (**b**) for the i-$Li_xCo_3O_4$ and h-$Li_xCo_{3-x}O_4$ series the spinel catalysts together with the bare Co_3O_4.

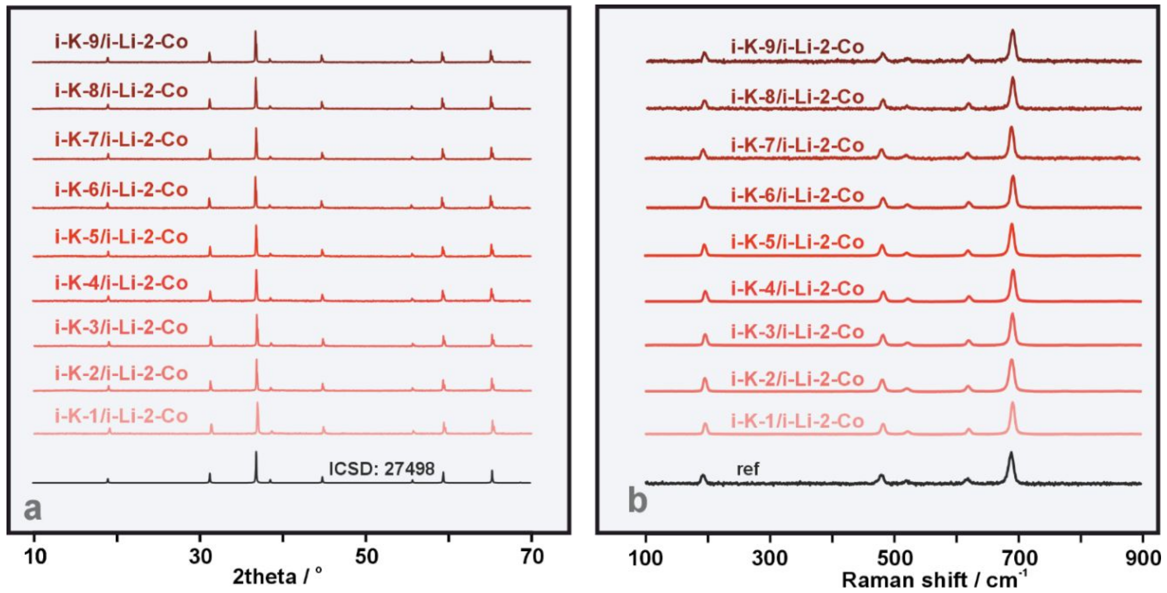

Figure S6: Powder X-ray diffraction patterns (**a**), and Raman spectra (**b**) for the i-K_y/$Li_{0.015}Co_3O_4$ series of the catalysts.

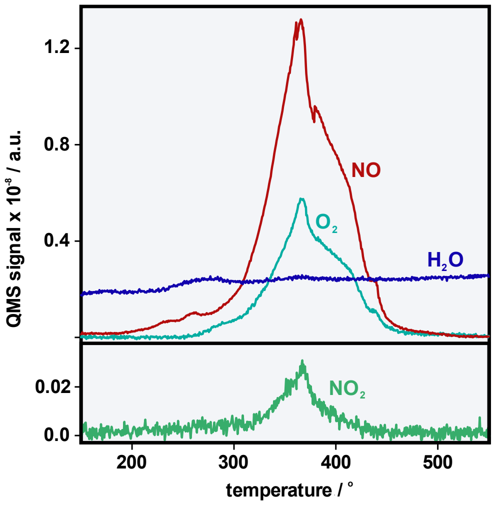

Figure S7. Evolution of NO, NO_2, O_2, and H_2O during calcination of the cobalt spinel nanocubes impregnated with $LiNO_3$.

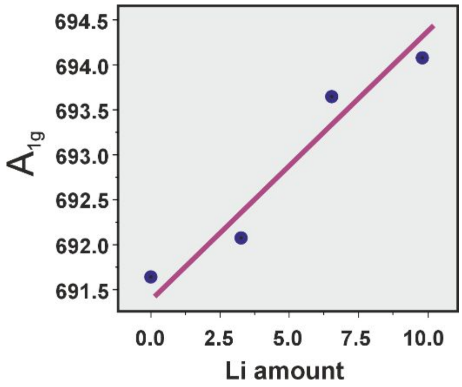

Figure S8. A shift of the A_{1g} Raman peak upon doping of the Co_3O_4 cubes with the increasing Li amounts.

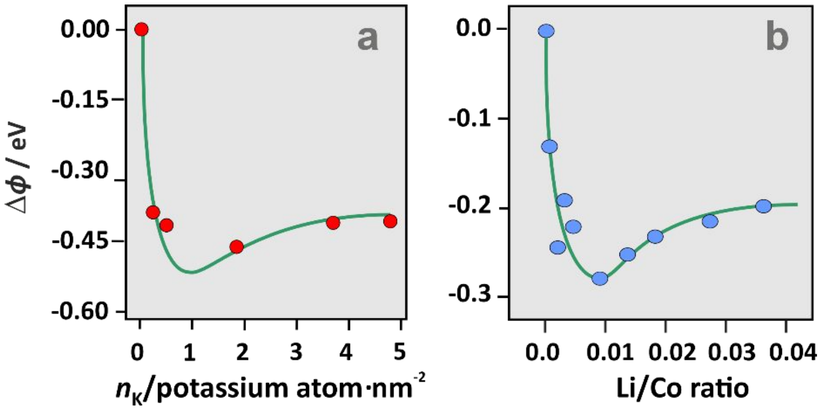

Figure S9. Work function changes for the spinel catalyst with the increasing areal concentrations of potassium, n_K, (**a**), and lithium loading, expressed as Li/Co ratio, (**b**), varying in a large range.

Chapter S4. Catalytic performance

Concisely, the decomposition of N_2O can be described by assuming a 3 step mechanism involving dioxygen evolution via surface diffusion of the charged monoatomic oxygen intermediates (Langmuir-Hinshelwood step, L-H)

$$N_2O_{(g)} + e \xrightarrow{k_1} N_{2(g)} + O^- \qquad \textbf{Eq. S1}$$

$$2O^- \xrightarrow{k_2} O_{2(g)} + 2e \qquad \textbf{Eq. S2}$$

$$2O^- \xleftarrow{k_{-2}} O_{2(g)} + 2e \qquad \textbf{Eq. S3}$$

or alternative formation of dioxygen via Elay-Riedel mechanism (E-R)

$$N_2O_{(g)} + O^- \xleftarrow{k_3} O_{2(g)} + N_{2(g)} + e \qquad \textbf{Eq. S4}$$

All these steps are of redox character, driven by the forth and back electron transfer between the catalyst surface and the reactant/intermediate adspecies. The corresponding rate equations can be formulated in the following way:

$$r_1 = k_1 p_{N2O}(1 - \theta) \qquad \textbf{Eq. S5}$$

$$r_2 = k_2\theta^2 \qquad \textbf{Eq. S6}$$

$$r_{-2} = k_{-2}(1 - \theta) \qquad \textbf{Eq. S7}$$

$r_3 = k_3 p_{N2O}\theta$ **Eq. S8**

The resultant reaction rate of N_2O decomposition can be described as $r_{N2O} = r_1 + r_3$, whereas k_2 encompasses successive oxygen diffusive recombination and desorption events associated with gradual transformation $2O^-_{(ads)} \xrightarrow{k_{rec}} O^-_{2(ads)} + e^- \xrightarrow{k_{des}} O_2 + e^-$, merged kinetically into $1/k_2 = 1/k_{rec} + 1/k_{des}$. These events depend strongly on the surface topology (diffusion), coverage (recombination and surface electrostatics), and position of the Fermi level for acceptance of electrons during successive oxidation of the anionic oxygen intermediates via back electron transfer. The E-R contribution is mainly related to the rigid oxygen intermediates (O-Co^T), associated with the exposed but distal surface tetrahedral cations. Due to the significantly higher activation energy, in comparison to the diffusive-recombination of the itinerant oxygen intermediates allied with the octahedral Co^O cations (1.28 vs 0.81 eV, respectively [3]), this pathway can be omitted in the first account.

The reaction rate $r_1 = k_1 p_{N2O}(1 - \theta)$ can alternatively be described by an empirical power low as: $r_{N2O} = kp_{N2O}{}^m$, where $m = \partial \ln r_{N2O} / \partial \ln p_{N2O}$). Deviation of m from an integer value (first-order kinetics) is due to the $\partial \ln(1 - \theta)/\partial \ln p_{N2O}$ term (see **Figure S12c**) that covers all mechanistic complexities arising from the more intricated oxygen evolution processes, that are sensitive to the surface topology. Conceivable scenarios of surface oxygen migration are schematically shown in the scheme below.

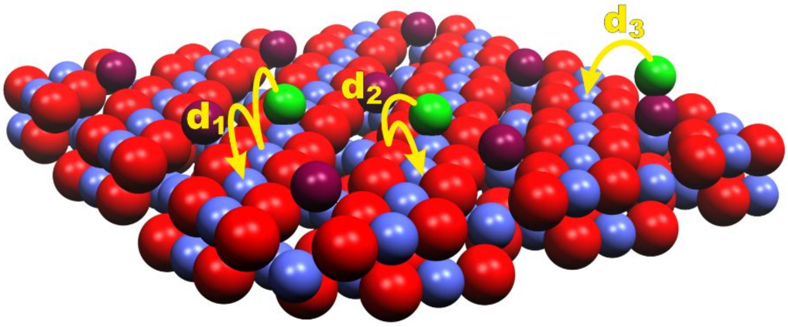

d_1 - diffiusion of the itinerant oxygen (marked green) intermediates along the truncated octahedral cobalt (Co^{oct}_{5c}, marked blue) rows
d_2 - mixed diffiusion of the itinerant oxygen intermediates along the truncated octahedral cobalt (Co^{oct}_{5c}) and O^{2-}_{surf} (red) rows
d_3 - diffiusion of the rigid oxygen intermediates from truncated tetrahedral cobalt (Co^{oct}_{5c}, purple) to octahedral (Co^{oct}_{5c}) centers

It should be emphasized, that the overall scheme of the N_2O decomposition reaction (reaction network) is maintained upon doping, and the latter modify essentially its energetic (Li, K) and impose steric constraints (K), modifying the course of the d_1 to d_3 diffusion pathways. As discussed in the main text in more detail, lithiation of cobalt spinels influences the activation energies by moving the Fermi level position, depending on the cobalt valence alternation that is controlled by the way the Li dopant is introduced (controlling the essentially the first step of the reaction, Eq. S1). The twofold role of potassium consists of beneficial reduction of surface potential facilitating electron transfer and unfavorable steric blocking of the surface recombination of the itinerant oxygen intermediates (Eq. S2 and S3).

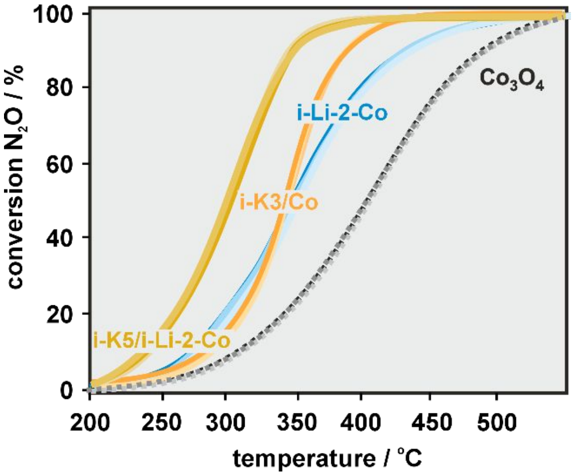

Figure S10. Reproducibility of the N_2O conversion profiles as a function of temperature for the bare, singly and doubly doped cobalt spinel catalysts. All experiments were performed 3-times. The obtained $X(T)$ vs T profiles are practically the same.

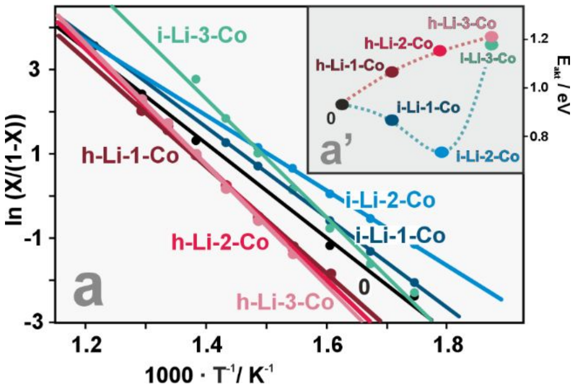

Figure S11: Arrhenius plots for N_2O decomposition over i-$Li_xCo_3O_4$ and h-$Li_xCo_{3-x}O_4$ samples (**a**), along with the corresponding activation energies **(a')**

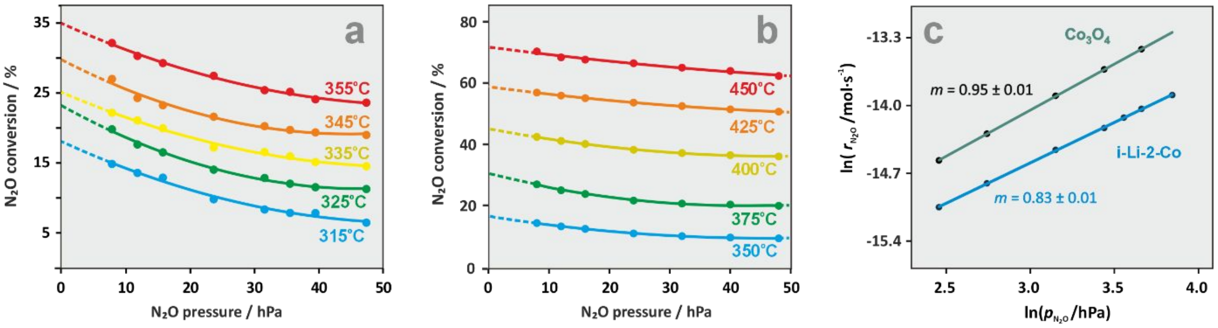

Figure S12. N_2O conversion profiles as a function of p_{N2O} pressure at various temperatures for the most active i-Li-2-Co (**a**) and the reference c-Co_3O_4 catalyst (**b**), along with the plot for determination of the apparent reaction order at the exemplary temperature 450 °C (**c**). The dotted lines indicate the conversion values obtained by the least square extrapolation to zero pressure.

In the case of bare Co_3O_4, the apparent reaction order is close to one (m = 0.95), confirming that N_2O dissociation induced by the interfacial electron transfer is rate-determining. For the most active among the lithiated samples (i-Li-2-Co catalyst), the apparent reaction order drops to m = 0.83 revealing an enhanced influence of the subsequent oxygen evolution step on the overall reaction rate. This can be accounted for by the shift of the Fermi level to higher energies that facilitates the forth electron transfer for N_2O dissociation (**Eq. S1**) and hindering the back electron transfer for oxidation of anionic oxygen intermediates (**Eq. S2**).

Chapter S5. Basic structural and electronic characterization of Co_3O_4

The basic structural and electronic characterization of Co_3O_4 oxide is summarized in **Table S1**. The calculated lattice parameter and *u* parameter are in line with experimental values [4] and correspond to the d_{CoO}–O and d_{CoT}–O distances of 1.974 and 1.926 Å, respectively [5]. The Bader charges and magnetic moments calculated for tetrahedral (Co^T) and octahedral (Co^O) cobalt cations indicate clearly that they exhibit 2+ and 3+ oxidation states, respectively. The arrangement of magnetic moment on the tetrahedral cations sublattice is in line with the experimentally confirmed antiferromagnetic ground state of cobalt spinel oxide [6].

Table S4. Basic structural and electronic characterization of Co_3O_4 oxide

Structural parameters of reference Co_3O_4				
Symmetry group	lattice const / Å	*u* parameter	$d_{CoT–O}$ / Å	$d_{CoO–O}$ / Å
Fd3m	8.149	0.263	1.974 Å	1.926 Å
Characteristics of constituting ions				
	Wycoff site	Bader charge / |e|	μ / μ_B	Formal oxidation state
Co^T	8a (1/8 occupancy)	1.349	2.61 / –2.61	2+
Co^O	16c (1/2 occupancy)	1.461	0.00	3+
O	32e (FCC lattice)	–1.06	0.01 / –0.01	2–

Chapter S6. Walsh diagram and potential energy surfaces of N_2O and N_2O^-

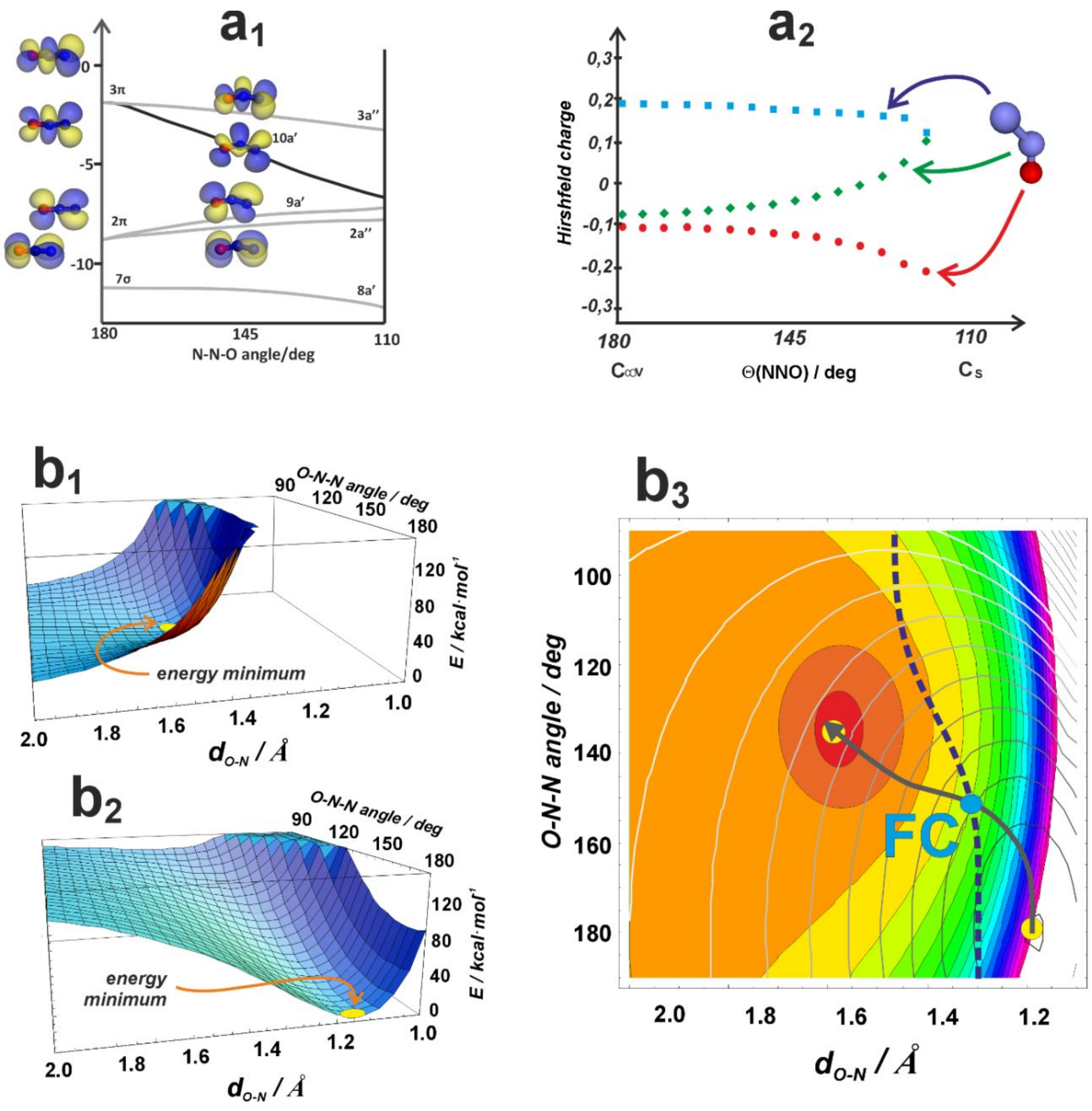

Figure S13. Walsh diagram of the N_2O molecule with the contours of the corresponding orbitals (**a$_1$**) together with partial charge redistribution upon the N_2O molecule bending (**a$_2$**). Potential energy surfaces for N_2O^- (**b$_1$**) and N_2O (**b$_2$**), and their overlay revealing the locus of the Frank-Condon point (**b$_3$**).

Chapter S7. DOS structure of the (100) surface of Co_3O_4, and pDOS profiles of the exposed truncated octahedral Co^{oct}_{5C} cations.

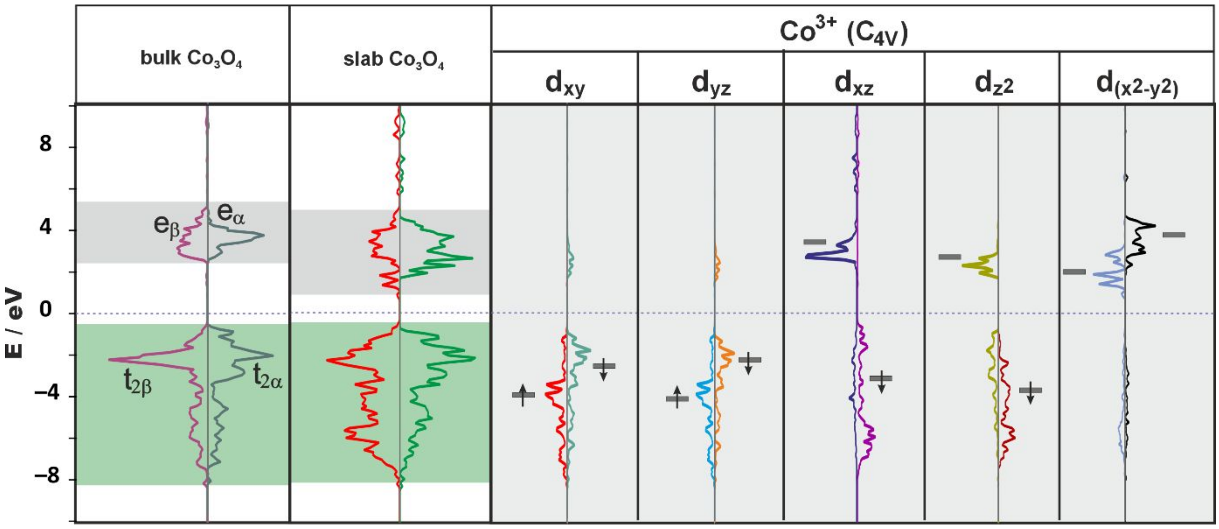

Figure S14. The DOS structure of the bulk Co_3O_4 and its (100) surface (left panels), together with the orbitally resolved partial DOS plots of exposed truncated octahedral Co^{oct}_{5C} cations (right panels)

Chapter S8. Energetic profiles for oxygen evolution on bare and Li-doped Co_3O_4

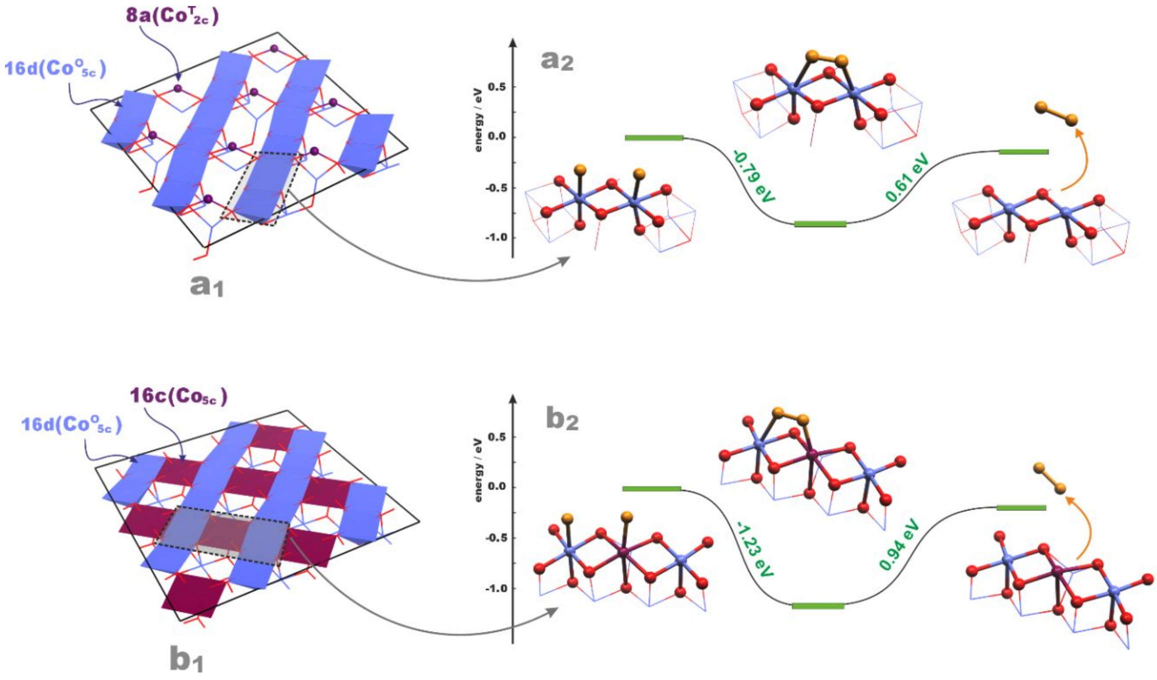

Figure S15. Energetic profiles for oxygen evolution on the 16d-16d (**a**) and 16d-16c (**b**) cobalt active sites, present in the bare Co_3O_4 and i-$Li_xCo_3O_4$ catalysts.

REFERENCES

[1] Zasada, F.; Piskorz, W.; Janas, J.; Gryboś, J.; Indyka, P.; Sojka, Z. Reactive Oxygen Species on the (100) Facet of Cobalt Spinel Nanocatalyst and their Relevance in $^{16}O_2/^{18}O_2$ Isotopic Exchange, deN_2O, and deCH_4 Processes-A Theoretical and Experimental Account. *ACS Catalysis* **2015**, *5*, 6879–6892.

[2] Zasada, F.; Stelmachowski, P.; Maniak, G.; Paul, J-F.; Kotarba, A.; Sojka, Z. Potassium Promotion of Cobalt Spinel Catalyst for N_2O Decomposition—Accounted by Work Function Measurements and DFT Modelling. *Catal. Lett.* **2009**, *127*, 126–131.

[3] Zasada, F.; Piskorz, W.; Janas, J.; Budiyanto, E.; Sojka, Z. Dioxygen Activation Pathways over Cobalt Spinel Nanocubes – From Molecular Mechanism into Ab Initio Thermodynamics and $^{16}O_2/^{18}O_2$ Exchange Microkinetics. *J. Phys. Chem. C* **2017**, *121*, 24128–24143.

[4] Stelmachowski, P.; Maniak, G.; Kaczmarczyk, J.; Zasada, F.; Piskorz, W.; Kotarba, A.; Sojka, Z. Mg and Al Substituted Cobalt Spinels as Catalysts for Low Temperature deN_2O – Evidence for Octahedral Cobalt Active Sites. *Appl. Catal. B* **2014**, *146*, 105−111.

[5] Liu, X.; Qiu, G.; Li, X. Shape-controlled Synthesis and Properties of Uniform Spinel Cobalt Oxide Nanocubes. *Nanotechnology* **2005**, *16*, 3035−3040.

[6] Shinde, V. R.; Mahadik, S. B.; Gujar, T. P.; Lokhande, C. D. Supercapacitive Cobalt Oxide (Co_3O_4) Thin Films by Spray Pyrolysis. *Appl. Surf. Sci.* **2006**, *252*, 7487−7492.
